# Affinity chromatography reveals direct binding of the GATA4–NKX2-5 interaction inhibitor (3i-1000) with GATA4

**DOI:** 10.1038/s41598-024-59418-4

**Published:** 2024-04-18

**Authors:** Mikael Jumppanen, Sini M. Kinnunen, Matej Zore, Mika J. Välimäki, Virpi Talman, Gustav Boije af Gennäs, Heikki J. Ruskoaho, Jari Yli-Kauhaluoma

**Affiliations:** 1 Drug Research Program, Division of Pharmaceutical Chemistry and Technology, Faculty of Pharmacy, University of Helsinki, Viikinkaari 5 E, (P.O. Box 56), FI-00014 Helsinki, Finland; 2 Drug Research Program, Division of Pharmacology and Pharmacotherapy, Faculty of Pharmacy, University of Helsinki, Viikinkaari 5 E, (P.O. Box 56), FI-00014 Helsinki, Finland

**Keywords:** Target identification, Cardiovascular biology

## Abstract

Heart failure is a serious medical condition with a poor prognosis. Current treatments can only help manage the symptoms and slow the progression of heart failure. However, there is currently no cure to prevent and reverse cardiac remodeling. Transcription factors are in a central role in various cellular processes, and in the heart, GATA4 and NKX2-5 transcription factors mediate hypertrophic responses and remodeling. We have identified compounds that modulate the synergistic interaction of GATA4 and NKX2-5 and shown that the most promising compound (**1**, **3i-1000**) is cardioprotective in vitro and in vivo. However, direct evidence of its binding site and mechanism of action has not been available. Due to the disordered nature of transcription factors, classical target engagement approaches cannot be utilized. Here, we synthesized a small-molecule ligand-binding pulldown probe of compound **1** to utilize affinity chromatography alongside CETSA, AlphaScreen, and molecular modeling to study ligand binding. These results provide the first evidence of direct physical binding of compound **1** selectively to GATA4. While developing drugs that target transcription factors presents challenges, advances in technologies and knowledge of intrinsically disordered proteins enable the identification of small molecules that can selectively target transcription factors.

## Introduction

Cardiovascular disease accounts for the majority of deaths and hospitalizations, healthcare expenditures, and loss of productivity in developed countries^1,2^. Heart failure is a condition in which the heart is unable to pump blood efficiently to meet the body’s needs. It affects more than 60 million people, is the most common cardiovascular cause for hospital admission of people older than 60 years of age and is associated with a poor prognosis^3^. Thus, delaying and preventing pathological myocardial remodeling, a key process in the development of heart failure, has become increasingly important in patients who are prone to heart failure. However, despite the best possible evidence-based drug treatment (including inhibitors of the renin–angiotensin–aldosterone system and β-blockers), morbidity and mortality rates remain high in patients with heart failure, and novel therapeutic strategies are necessary to prevent and reverse cardiac remodeling^4^.

Most drug discovery projects have been based on the molecular target hypothesis, driven by advances in genomics and molecular biology^5^. Phenotypic screening assays, which now contribute more significantly than target-based approaches to the discovery of first-in-class small-molecule drugs, further expand drug target diversity^6^. Therefore, the identification of targets in phenotypic drug discovery projects is considered a crucial component of project progression and prioritization by most large pharmaceutical companies^5^. Recently, we reported the identification of small molecules that either inhibit or enhance the GATA4–NKX2-5 transcriptional synergy^7–9^. GATA4 and NKX2-5 are the master transcription factors in the heart and are required for cardiogenesis^10,11^. In the adult heart, GATA4 and NKX2-5 are critical regulators of cardiac remodeling and there is evidence for a functional role of GATA4–NKX2-5 interaction in mediating cardiac gene activation and cardiomyocyte hypertrophy^10,12^. The most potent inhibitor (compound **1**, **3i-1000**) of GATA4–NKX2-5 interaction reduced cardiomyocyte hypertrophic response in vitro^7^, ameliorated hypertrophic signaling in vivo^13^, and improved cardiac function in vivo^13,14^ in experimental models of myocardial infarction and hypertension.

Our previous study suggested that compound **1** may mediate its effect on the transcriptional synergy of GATA4 and NKX2-5 through direct binding to GATA4 since it significantly decreased phenylephrine-induced GATA4 Ser-105 phosphorylation in cardiomyocytes^7^. Therefore, the aim of the present study was to investigate the binding of compound **1** to GATA4 or NKX2-5 to explore its mode of action. Conventional ligand engagement methods require a large quantity of pure recombinant protein which usually consists of only one domain or a part of the protein. Here, we aimed to study ligand binding with full-length GATA4 protein produced in mammalian cells to ensure proper protein folding and post-translational modifications and to avoid protein purification processes. The binding of compound **1** to GATA4 was examined by using affinity chromatography and molecular modeling docking studies to our previously published homology model^15^. Affinity chromatography is conventionally used for protein purification^16^, but is also widely applied for identifying targets of biologically active compounds^17,18^. Typically, in affinity chromatographic target identification of small molecules, the ligand is covalently bound to a linker molecule and subsequently attached to an immobile matrix such as Sepharose®. Hydrophilic polyethylene glycol (PEG) linkers are commonly used to reduce unspecific binding to lipophilic proteins compared to alkyl linkers^17^. Moreover, established structure–activity relationship (SAR) knowledge is required for determining the correct attachment site of the linker so that the immobilized ligand retains its binding affinity to the target^18^.

## Results

### Synthesis of pulldown probes

**Figure 1:**
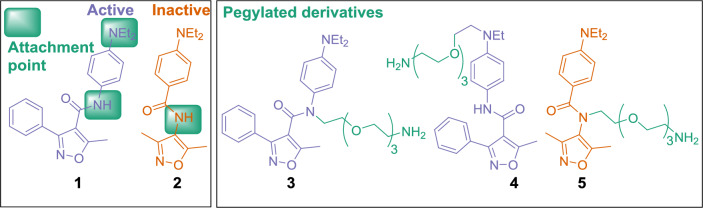
Active and inactive compounds **1** and **2**, and their synthetic derivatives **3–5** with PEG_3_ linkers for affinity chromatography.

To design an affinity chromatography pulldown probe, the attachment chemistry, linker design, matrix selection, and controls were considered. In our previous studies,^7,9^ we identified that the essential structural features for potent compounds in a GATA4 and NKX2-5 transcriptional synergy assay are the relatively rigid linker with a hydrogen bond acceptor (e.g., amide bond) and a hydrogen bond acceptor, donor, or otherwise polar group in the upper part (e.g., aniline ring in the hit compound **1**), implicating that the oxygen atom of the amide bond and the dialkylamino group are indispensable features for the activity of compound **1**. However, based on the modeling, these sites are most likely exposed to solvent. Therefore, the amide nitrogen atom and the alkyl chain of the diethylamino group of the compound **1** were selected as attachment sites for the PEG_3_ linker (Fig. 1). For control experiments, the PEG_3_ linker without compound and inactive compound **2** were chosen. Compound **2** possesses the substructural similarity to compound **1** with a reversed amide bond and without a phenyl ring, although it is inactive in the GATA4–NKX2-5 transcriptional synergy assay^9^.

**Figure 2:**
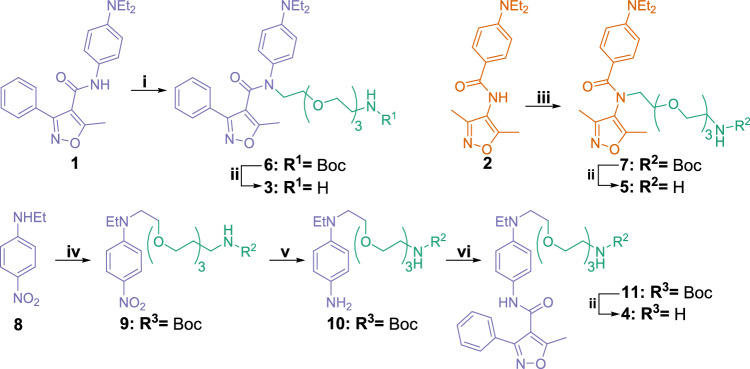
Synthesis of compound **1** derivatives and an inactive control compound **2** with PEG_3_ linkers. Reagents and conditions: (i) KH (30 w/w %, mineral oil), Boc-PEG_3_-Br, THF, rt, 2 d; (ii) 4 M HCl in 1,4-dioxane, 0 °C → rt, 2–4 h; (iii) KH (30 w/w %, mineral oil), Boc-PEG_3_-Br, DMF, rt → 80 °C, 72 h; (iv) NaH (60 w/w %, mineral oil), Boc-PEG_3_-Br, DMF, 0 °C → rt, 1 d; (v) H_2_, Pd/C, rt, 1 d; (vi) 5-methyl-3-phenylisoxazole-4-carboxylic acid, HATU, Et_3_N, DMF, rt, 3 d.

**Figure 3:**
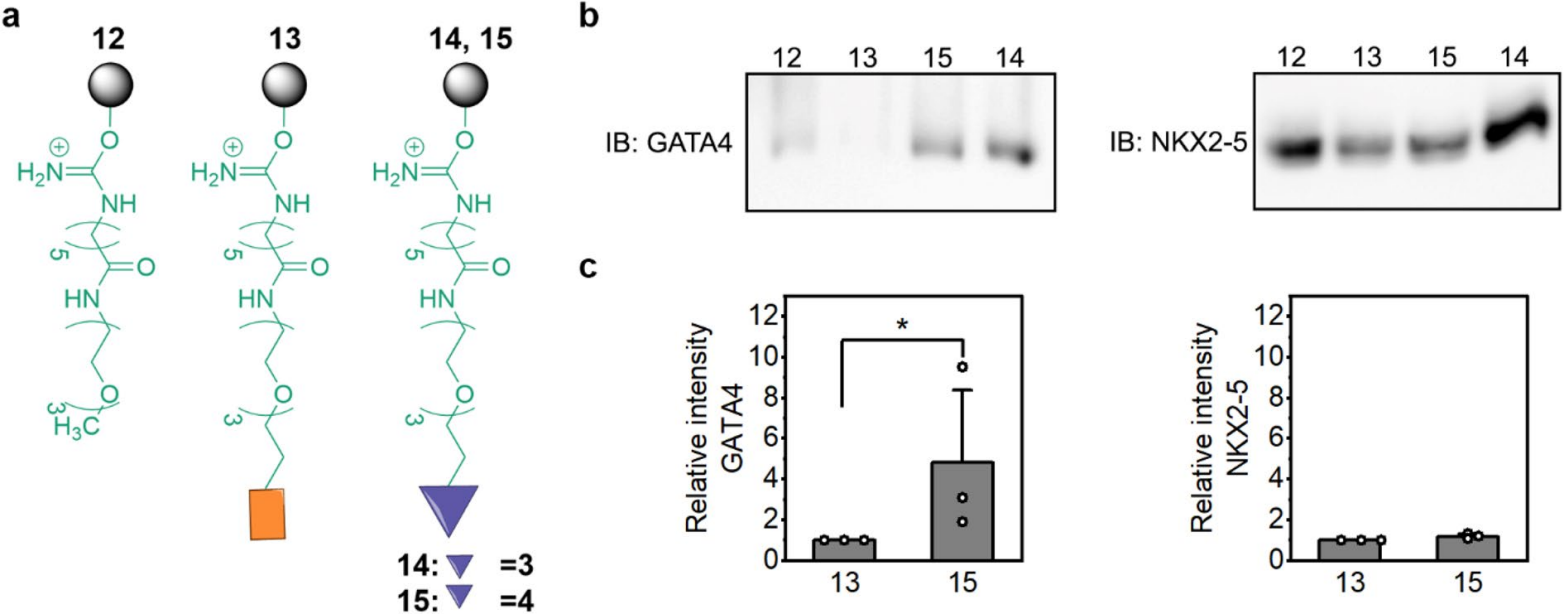
(**a**) Binding of GATA4 or NKX2-5 proteins to pulldown probes (**12**–**15**). (**b**) Affinity chromatography was performed with total protein lysates overexpressing GATA4 protein or NKX2-5 protein together with negative control pulldown probes **12** (PEG_3_ linker) and **13** (inactive ligand) or pulldown probes **14** and **15** (active ligands). GATA4 and NKX2-5 antibodies were used to visualize bound proteins. The images present the overview of the binding experiments, which were repeated 3–4 times for each probe, except the GATA4 binding with probe **12** two times. The whole blot images are presented in Supplementary Fig. S3 and S4. (**c**) The binding intensity of inactive ligand (**13**) compared to active ligand (**15**) was quantified. The results show the average, + SEM and single data points of each individual experiment (n = 3). *p < 0.05 as indicated (Mann–Whitney U test).

Synthesis of the pegylated derivatives **3** and **4** of compound **1** and the derivative **5** of the inactive control compound **2** is described in Fig. 2. Compounds **3** and **5** were obtained via a straightforward S_N_2 reaction of Boc-PEG_3_-Br and compounds **1** and **2**, using potassium hydride as a base to deprotonate the amide, followed by the Boc removal in a 4 M solution of HCl in 1,4-dioxane. The preparation of compound **4** required a multistep synthesis starting from *N*-ethyl-4-nitroaniline (**8**). Boc-PEG_3_-Br was attached to compound **8** with sodium hydride-mediated removal of hydrogen of the secondary amino group followed by S_N_2 reaction to give pegylated compound **9**. The nitro group of the compound **9** was reduced to the primary amino group of the compound **10** with catalytic hydrogenation (Pd/C). The subsequent amide coupling reaction of compound **10** with 5-methyl-3-phenylisoxazole-4-carboxylic acid in the presence of *N*-[(dimethylamino)-1*H*-1,2,3-triazolo-[4,5-*b*]pyridin-1-ylmethylene]-*N*-methylmethanaminium hexafluorophosphate *N*-oxide (HATU) and triethylamine in *N*,*N*-dimethylformamide gave compound **11**. Removal of the Boc group with a 4 M solution of HCl in 1,4-dioxane gave free amine **4**. Pegylated compounds **3–5** and the sole PEG_3_-linker with a methyl end were covalently immobilized via a stable amide bond to *N*-hydroxysuccinimide-activated Sepharose® to give pulldown probes **12–15** (Fig. 3a). Immobilization of the compounds was verified with Fourier-transform infrared spectroscopy (FTIR) by comparing FTIR spectra of the immobilized compound and control Sepharose^®^, which went through similar immobilization procedure. We consistently found the FTIR stretching vibrational peak in the aromatic region for the pulldown probes with active ligands (**14**: 1518 cm^−1^, **15**: 1516 cm^−1^). For the negative control pulldown probe **13** (inactive ligand) we found aromatic and ether vibrational peaks (**13**: 1603 cm^−1^ and 1270 cm^−1^). For the negative control pulldown probe **12** (PEG_3_ linker) we were unable to find clearly distinguishable vibrational peak compared to the Sepharose® control. Furthermore, pegylated compound **3** was found to be inactive in GATA4–NKX2-5 transcriptional synergy cell assay,^7,9^ whereas pegylated compounds **4** and **5** have not been tested. Pegylated compounds with free primary amines are ionized in luciferase assay conditions, which most likely drastically reduces membrane permeability and prevents activity.

### Protein binding to pulldown probes

The pulldown probes (**12**–**15**) were used in affinity chromatography experiments to detect possible binding of GATA4 or NKX2-5 to them. The schematic representation of affinity chromatography is shown in Fig. 3a. The protocol for affinity chromatography was modified from the immunoprecipitation protocol that we have successfully used to study GATA4–NKX2-5 interaction^15^. GATA4 and NKX2-5 proteins were overexpressed separately in COS-1 cells and the total cell lysate was extracted in non-denaturing conditions that preserve protein conformation, which is important for compound binding. After overnight incubation of protein lysate with the pulldown probe, the samples were washed several times to remove unbound proteins (Supplementary Fig. S1,S2). Finally, the samples were boiled in SDS sample buffer to elute bound proteins, which were then resolved by SDS-PAGE and immunoblotted with GATA4 or NKX2-5 antibodies (Fig. 3b). The whole blot images are presented in Supplementary Fig. S3,S4. As shown in Fig. 3b, GATA4 bound to pulldown probes with active ligands **14** and **15**, but not to the negative control pulldown probe **13** with an inactive ligand. A small amount of unspecific GATA4 binding to negative control pulldown probe **12** (PEG_3_ linker) was also observed. NKX2-5, however, exhibited comparable binding to the negative control pulldown probe **13** (inactive ligand) and the pulldown probe **15** (active ligand). Furthermore, as NKX2-5 binding to the PEG_3_ linker **12** was even more pronounced, the binding of NKX2-5 was likely unspecific. As PEG is a synthetic, hydrophilic, and biocompatible polyether, significant protein binding to PEG_3_ linker without compound (**12**) was anticipated. The quantification of relative binding intensities shows significantly increased GATA4 binding to active ligand (**15**) compared to inactive ligand (**13**) (Fig. 3c). Biological variability between experiments is related to the experimental protocol, as each protein sample for affinity chromatography was prepared separately.

### Compound 1 stabilizes GATA4 protein

**Figure 4:**
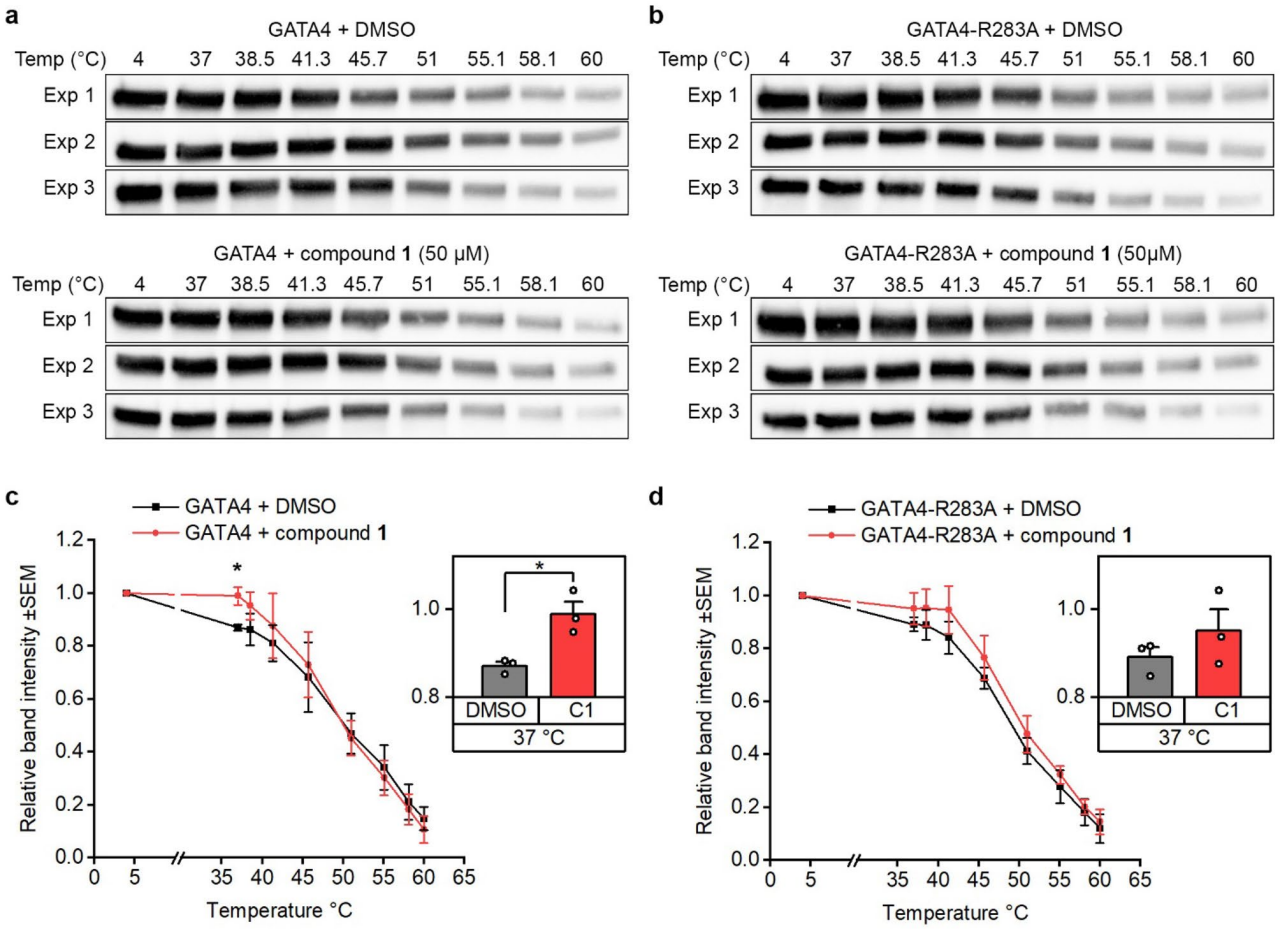
CETSA experiments showing ligand engagement to wild type GATA4. Adherent COS-1 cells expressing either wild type (**a**) or mutated GATA4 (**b**) were exposed to 0.1% DMSO or 50 µM of compound **1** for 1 h at 37 °C. The cells were trypsinized, non-denatured total proteins were extracted, and the sample aliquots were exposed to temperature gradient. Unfolded GATA4 proteins were detected by western blot and band intensities were quantified (**c**, **d**). The results are shown as the average of 3 independent experiments ± SEM, bar graphs show each individual datapoint + SEM at 37 °C, *p < 0.05 (Independent samples t-test).

The ligand engagement of compound **1** to GATA4 was further verified with cellular thermal shift assay (CETSA) (Fig. 4a–d). COS-1 cells were transfected for 6 h with native GATA4 or GATA4-R283A mutation plasmid^15^, and cultured in the normal culturing medium for approximately 20 h before exposing adherent cells to compound **1** (50 µM) or 0.1% DMSO for 1 h in cell culture incubator. Native proteins from whole cell lysates were set under a thermal gradient and after the removal of aggregated proteins, the samples were analyzed by western blot (Fig. 4a,b) and quantified (Fig. 4c,d). The results show that the melting temperature of GATA4 is relatively low, approximately 37 °C (Fig. 4c). Compound **1** treatment induced GATA4 protein stabilization at 37 °C compared to DMSO-treated group (*p* < 0.05, Independent samples t-test, SPSS). We have previously reported that the mutation R283A in GATA4 C-terminal zinc finger inhibits GATA4 protein interaction with NKX2-5 and affects GATA4 transcriptional activity and DNA binding^15^. GATA protein mutated at R283A inhibited but did not completely abolish stabilization induced by compound **1** (Fig. 4d).

### Compound 1 inhibits GATA4–NKX2-5 protein–protein interaction

**Figure 5:**
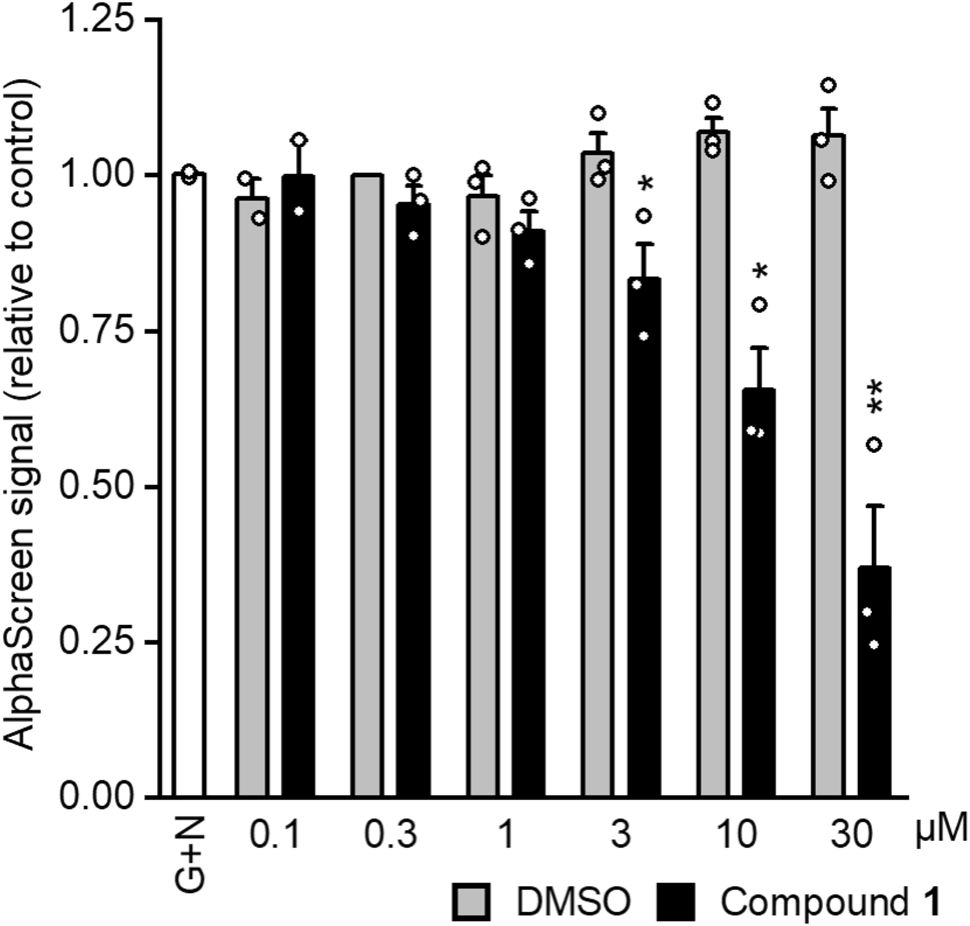
Compound **1** inhibits GATA4–NKX2-5 interaction concentration-dependently. GATA4-V5 tag and NKX2-5-SH tag whole cell lysates were combined and incubated with compound **1** or DMSO dilution series for 1 h before adding the AlphaScreen microbeads. Results were adjusted to 0.3 µM DMSO control and are expressed as the mean + STDEV of 3 independent experiments (2 experiments for 0.1 µM). G + N denotes GATA4–NKX2-5 (n = 1). *p < 0.05, **p < 0.01 versus control group (Independent samples t-test).

We have previously shown that compound **1** inhibits GATA4–NKX2-5 interaction by immunoprecipitation and by luciferase assays,^7^ and used here AlphaScreen method to further prove the action of compound **1** on GATA4–NKX2-5 interaction. GATA4 protein with C-terminal V5-tag and NKX2-5 with N-terminal SH-tag were produced in COS-1 cells separately and the whole cell lysates were extracted in non-denaturing conditions. Both proteins were combined in the same wells at appropriate concentrations together with compound **1** or DMSO dilution series and incubated for 1 h at 4 °C before the addition of V5-acceptor beads and Strep-Tactin donor beads (See Supplementary information and Figures S5–S7 for method optimization). GATA4 and NKX2-5 interaction produced an Alpha signal, which decreased concentration-dependently with compound **1**. This confirms that compound **1** inhibits protein–protein interaction (Fig. 5).

### Docking

The affinity chromatography and CETSA results do not indicate the mode of action and site of the ligand binding. Therefore, the binding preference of compound **1** was further evaluated with molecular modeling and docking studies. Due to their primary function in decoding the genome, transcription factors interact with DNA and multiple co-factors simultaneously in a context-dependent manner. In comparison to other protein classes, transcription factors have typically a high intrinsic structural flexibility and disorder that maximizes the specificity of promiscuous interactions^19,20^. Thus, we ignored the use of previously resolved unbound NMR structure of GATA4 from *Homo sapiens* as a model (Protein Data Bank; 2M9W) and concentrated on the existing DNA-bound structure of GATA-protein (Protein Data Bank; 3DFX) as a structurally more stable and functionally relevant protein template^15,21^. It is broadly recognized that the quality of homology models may vary, and protein models alone should not be used to generate any decisive conclusions.

**Figure 6:**
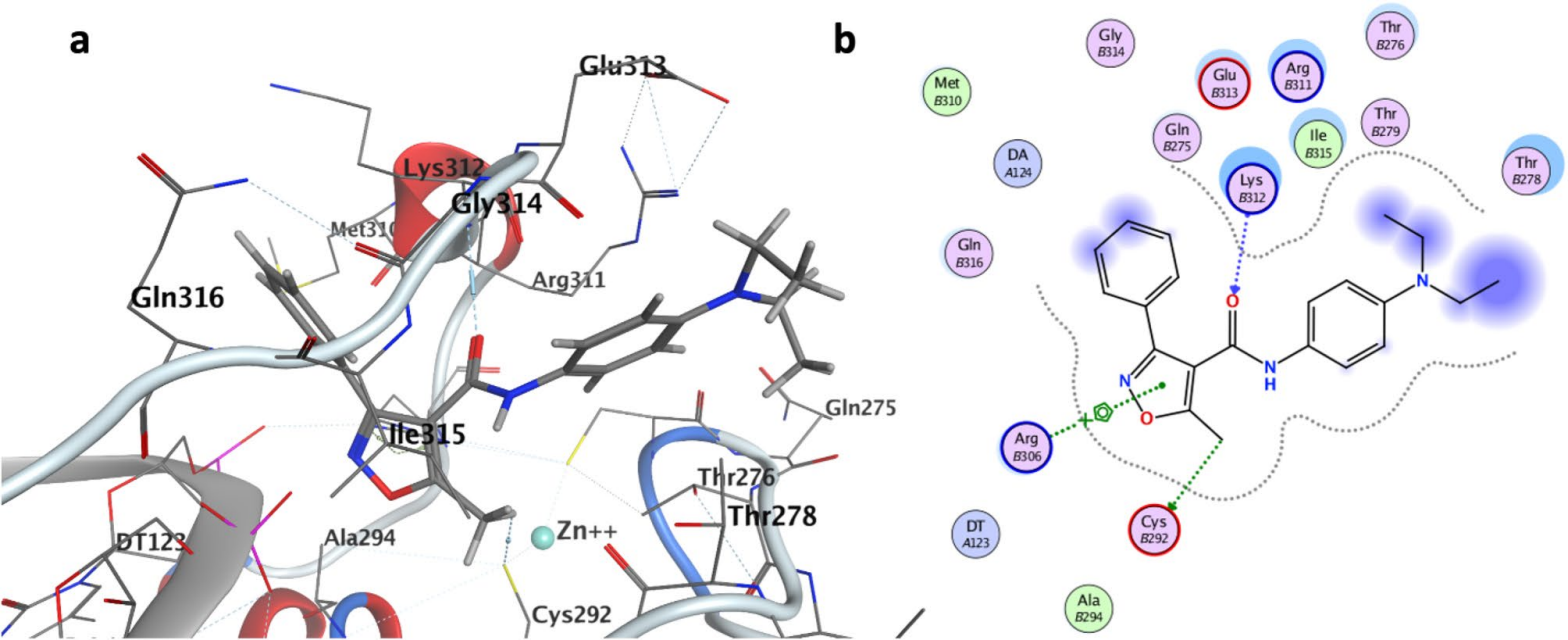
Proposed binding mode of compound **1** in C-terminal zinc finger of GATA4 resolved by docking experiments (**a**). Ligand interaction diagram of compound **1** illustrating the important amino acids relevant for GATA4 binding (**b**).

The proposed binding mode of compound **1** to GATA4 and ligand interaction diagram of compound **1** are presented in Fig. 6a and b, respectively. Docking of compound **1** and inactive derivative **2** into the protein model of GATA4 generated the affinity scores of − 8.06 and − 6.98 kcal/mol, respectively. Interestingly, the ligand binding scores for the active compound **1** and the inactive compound **2** suggested a meaningful shift in protein binding affinities and are thus in line with our current experimental findings. As shown in the ligand interaction diagram (Fig. 6b), the most important amino acids mediating the compound binding are the zinc finger coordinating Cys292 and positively charged residues Arg306 and Lys312.

## Discussion

The cardiac remodeling process is regulated by multiple pathways involving cross-talk with signaling components and various transcription factors eventually leading to alterations in gene expression^22^. Due to their central role in gene regulation, transcription factors are potential and attractive therapeutic targets^23^. However, developing small molecules that target transcription factors is challenging because they lack enzymatic activity, are predominantly intrinsically disordered, and lack classical well-formed small-molecule binding pockets^19^. For a long time, transcription factors (other than nuclear receptors) and their protein–protein interactions (PPI) were considered as undruggable targets^24–27^. All transcription factors have a DNA-binding domain and effector domain that recruits various transcriptional ‘collaborators’ to regulate chromatin accessibility, interaction with co-activators/co-repressors, and general transcriptional machinery. Many transcription factors also contain one or more regulatory domains, which serve to regulate dimerization, nuclear transport, and functional activity^26^. Of these domains, DNA-binding domains are typically more structured than the effector domains, which are disordered when unbound to partner proteins^26^. The advances in structural characterization, basic biological insights, and ligand design strategies have made it possible to identify chemical probes that target transcription factors^26^.

We used here affinity chromatography for ligand engagement studies. Affinity chromatography is a powerful technique to isolate and purify proteins based on their interactions with specific ligands or binding partners^17,18^. The advantage of affinity chromatography is that the full-length target protein can be produced in mammalian cells, which enables proper protein folding and post-translational modifications, and pulled down directly from the whole cell lysate with an immobilized ligand. To study compound **1** binding to GATA4, affinity chromatography was used for several reasons. Initially, we reported by using co-immunoprecipitation and luciferase reporter assays that compound **1** affects GATA4–NKX2-5 interaction^7^. However, efforts to demonstrate direct physical binding of the compound to a target turned out to be challenging. We found that C-terminal zinc finger of GATA4 is structurally unstable and sensitive to lose its ordered native conformation in vitro. During the NMR-studies, both ^15^N- and ^13^C-labeled C-terminal zinc finger domains of human GATA-4 protein (residues 265–330) were successfully produced in *Escherichia coli* culture. However, the time-consuming purification procedure and exposure to DMSO caused the time-dependent change in GATA4-protein fold that was confirmed by NMR (data not shown). Despite these challenges, recombinant protein of GATA4 C-terminal zinc-finger has been successfully produced and resolved with NMR by the Northeast Structural Genomics Consortium (PDB, 2M9W).

Furthermore, our mutational studies and molecular modelling studies on GATA4 protein dynamics showed that alterations in the packing of C- and N-terminal zinc fingers interfere GATA4–NKX2-5 physical interaction^15^. Thus, for ligand engagement studies the use of whole GATA4 protein would be eligible. For microscale thermophoresis (MST), a smaller amount of pure protein is required and there is a commercially produced human recombinant GATA4 protein available. However, for the MST method the protein needs to be labeled with a fluorescent dye which forms a covalent bond with primary amines (lysine residues). Either the labeling process or the low concentration of GATA4 protein after the labeling procedure yielded low-quality protein samples. This was detected as protein aggregates in MST measurements and GATA4 protein degradation detected by western blot (data not shown). Therefore, affinity chromatography instead of MST was used. Additionally, our previous findings indicated that the compounds either did not affect, or only slightly inhibited (not significant) the DNA binding activities of GATA4 and NKX2-5^7,9^. The observed effects of these small molecules on the transcriptional synergy driven by GATA4 and NKX2-5 are unlikely to be attributable to either inhibition or activation of GATA4 or NKX2-5 DNA binding. Furthermore, the possibility that the compound binds to other GATA proteins in addition to GATA4 cannot be ruled out, presenting an intriguing avenue for future research.

For the affinity chromatography experiments, we first prepared pulldown probes **14**–**15**, consisting of active compound **1** immobilized to Sepharose® matrix via triethylene glycol (PEG_3_) linker. To ensure that the observed binding interactions are specific and not due to non-specific interactions, we synthesized a control pulldown probe **13** consisting of functionally inactive compound **2** attached to Sepharose® via PEG_3_ linker and a pulldown probe **12** consisting of only PEG_3_ linker attached to Sepharose®. A prerequisite for a successful use of affinity chromatography is to identify a site for linker attachment that can tolerate variations without significant loss of affinity to the target protein. SAR needs to be defined to determine which functional groups of a molecule are essential for its activity, and which groups can be used to attach a compound to a matrix^17^. We selected the amide nitrogen atom and the alkyl chain of the diethylamino group of compound **1** as attachment sites, as these two structural features were previously identified as non-essential for the activity. Additionally, these two sites enabled a simple attachment of the linker through 2-4-step synthesis. Another important consideration for affinity chromatography is the choice of a linker which is used to covalently attach the ligand to a solid matrix. The attachment of a linker to the ligand provides a functional group necessary for the immobilization of a ligand to a solid matrix. Moreover, it is also mandatory to prevent steric obstructions between the matrix and the targeted proteins. Several different types of linkers have been reported, such as alkyl groups, polyethylene glycol groups, peptides, divalent epoxides, and tartaric acid^17^. Among them, the hydrophilic polyethylene glycol spacers, especially di- and triethylene glycol, are most often the linkers of choice. Finally, the affinity probes were immobilized onto a solid matrix, and in our case, we chose *N*-hydroxysuccinimide activated Sepharose®. This type of pre-activated agarose enables the formation of stable amide bonds with primary amines. Successful attachment of the affinity probes to the Sepharose® was confirmed with FTIR.

Immobilized compounds were used in the same affinity chromatography conditions that have been previously utilized for GATA4 and NKX2-5 interaction studies^7,15^ and which maintain the protein conformation. Furthermore, the pulldown assay was validated to ensure proper washing of unbound proteins, and the identity of the target protein was confirmed by western blotting. Interestingly, both active compounds exhibited more binding to GATA4 than the inactive ligand. On the other hand, all the tested compounds exhibited similar binding to NKX2-5, implying unspecific binding. The binding to GATA4 was further confirmed by CETSA method, where compound **1** stabilized significantly the native GATA4 protein at 37 °C but had less effect on mutated GATA4 protein. It is noteworthy that a single mutation in GATA4 is unlikely to fully inhibit the stabilization of GATA4 under these experimental conditions. Furthermore, the intracellular concentration of free GATA4, which is not bound to chromatin, is low (unpublished observation of S.M. Kinnunen). Therefore, to perform affinity chromatography and CETSA studies, GATA4 was overexpressed. Thus, under these experimental conditions, excessive quantity of protein is present in comparison to ligand whose concentration cannot be increased due to aggregation and limitations in the experimental setup e.g., DMSO concentration. This affects the ligand binding sensitivity especially in CETSA. On the other hand, excessive target protein amount/concentration does not affect the detection sensitivity of affinity chromatography when the ligand is used as immobilised bait. Finally, AlphaScreen findings, not reported earlier, provide additional experimental evidence that compound **1** inhibits GATA4–NKX2-5 interaction.

The recent advances in biophysical understanding of intrinsically disordered transcription factors along with the development of experimental techniques that are highly suited to protein characterization have fostered the identification of transient pockets and their interactions with ligands^26^. This progress has enabled the design of small molecules targeting the ‘undruggable’ transcription factors. However, the target engagement and selectivity evaluation approaches for these challenging protein targets are still imperfect. The classical methods generally expect that the target proteins are structurally quite stable. Here, we synthesized a pulldown probe and used the affinity chromatography method and CETSA to show the first evidence of a direct physical interaction of GATA4–NKX2-5 interaction inhibitor (compound **1**) with GATA4 protein. Furthermore, AlphaScreen results together with our previous data confirm that compound **1** inhibits GATA4–NKX2-5 interaction.

## Methods

### Syntheses and characterization of compounds

#### General information

All reactions were conducted using commercially available starting materials and reagents. All chemicals, solvents and anhydrous solvents used in the synthesis of the novel compounds were acquired from Sigma-Aldrich (Schnelldorf, Germany), Acros Organics (Morris, New Jersey, USA), BroadPharm (San Diego, CA, USA), Honeywell (Bucharest, Romania) and were used without further purification. All moisture sensitive reactions were performed in flame-dried glassware under an inert argon atmosphere. The progress of chemical reactions was monitored by thin-layer chromatography on 0.2-mm silica gel plates (silica gel 60, F254, Merck KGaA, Darmstadt, Germany) and visualized by UV light or ninhydrin stain (1.5% by weight in ethanol), when applicable. Column chromatography was performed with automated Biotage high performance flash chromatography Sp4-system (Uppsala, Sweden) using a 0.1-mm path length flow cell UV-detector/recorder module (fixed wavelength 254 nm) and the indicated mobile phase. Nuclear magnetic resonance spectra (^1^H NMR and ^13^C NMR) were recorded on a Bruker Ascend 400—Avance III HD NMR spectrometer (Bruker Corporation, Billerica, MA, USA), ^1^H NMR spectra at 400 MHz and ^13^C NMR spectra at 101 MHz. Chemical shifts (*δ*) are reported in parts per million (ppm) relative to the NMR solvent signals (CDCl_3_ 7.26 and 77.16 ppm, DMSO-*d*_6_ 2.50 and 39.50 ppm for ^1^H and ^13^C NMR, respectively). When necessary, two-dimensional NMR experiments (COSY, NOESY, gHSQC, gHMBC) were conducted to support structure determination. Multiplicities are indicated by s (singlet), d (doublet), dd (doublet of doublets), t (triplet), q (quartet), sept (septet). The additional abbreviation “br” indicates a broad signal, such as br s (broad singlet). Multiplets (m) are reported as a range of ppm values. Coupling constants *J* are quoted in hertz (Hz). IR spectra were measured using Vertex 70 FTIR spectrometer (Bruker Corporation, Billerica, MA, USA). Exact mass and purity of synthesized compounds were confirmed by LC–MS analyses with a Waters Acquity® UPLC system (Waters, Milford, MA, USA) equipped with an Acquity UPLC® BEH C18 column (1.7 μm, 50 mm × 2.1 mm, Waters, Ireland), an Acquity PDA detector and a Waters Synapt G2 HDMS mass spectrometer (Waters, Milford, MA, USA) via an ESI ion source in positive mode. High resolution mass (HRMS-ESI) data was reported for the molecular ions [M+H]^+^.

#### 4-(Diethylamino)-*N*-(3,5-dimethylisoxazol-4-yl)benzamide (2)

4-(Diethylamino)benzoic acid (0.200 g, 1.04 mmol), 3,5-dimethylisoxazol-4-amine (0.139 g, 1.24 mmol, 1.2 equiv) and *N*-[(dimethylamino)-1*H*-1,2,3-triazolo-[4,5-*b*]pyridin-1-ylmethylene]-*N*-methylmethanaminium hexafluorophosphate *N*-oxide, HATU (0.511 g, 1.35 mmol, 1.3 equiv) were dissolved in dry DMF (8 mL). *N,N*-Diisopropylethylamine (DIPEA; 0.36 mL, 2.0 equiv) was added and the reaction mixture was stirred at room temperature for 23 h. The temperature was increased to 80 °C and the stirring was continued for additional 5 h. After a total of 28 h, water was added, and the aqueous phase was extracted with Et_2_O. The combined organic phases were dried with anhydrous Na_2_SO_4_, filtered, and concentrated on rotary evaporator. Chromatography on silica gel (EtOAc/*n*-heptane 20% → 80%) gave compound **2** (0.180 g, 61%) as a white solid. ^1^H NMR (400 MHz, CDCl_3_) *δ* 7.76 (d, *J* = 8.8 Hz, 2H), 7.19 (s, 1H), 6.65 (d, *J* = 8.8 Hz, 2H), 3.42 (q, *J* = 7.1 Hz, 4H), 2.32 (s, 3H), 2.19 (s, 3H), 1.20 (t, *J* = 7.1 Hz, 6H). ^13^C NMR (101 MHz, CDCl_3_) *δ* 166.4, 163.9, 158.3, 129.4, 118.8, 114.1, 110.6, 44.6, 12.6, 11.3, 10.0 ppm. HRMS calc. for C_16_H_22_N_3_O_2_ [M+H]^+^ 288.1712, found 288.1711.

#### *N*-[2-[2-[2-(2-Aminoethoxy)ethoxy]ethoxy]ethyl]-*N*-[4-(diethylamino)phenyl]-5-methyl-3-phenylisoxazole-4-carboxamide (3)

To a stirred solution of *tert*-butyl [2-(4-(diethylamino)phenyl]-1-(5-methyl-3-phenylisoxazol-4-yl)-1-oxo-5,8,11-trioxa-2-azatridecan-13-yl)carbamate **6** (18.3 mg, 0.0293 mmol) in 1,4-dioxane (0.5 mL), a 4 M solution of HCl in 1,4-dioxane (0.500 mL, 2.00 mmol) was added dropwise at 0 °C. After 10 min the reaction mixture was let warm to room temperature and stirring was continued for 2 h. Solvents were evaporated and a saturated aqueous solution of NaHCO_3_ was added. The aqueous phase was extracted with EtOAc. The combined organic phases were dried with anhydrous Na_2_SO_4_, filtered, and evaporated on rotary evaporator. Chromatography on silica gel (EtOAc/EtOH 3:1) gave compound **3** (9.00 mg, 59%) as a yellowish oil. ^1^H NMR (300 MHz, CDCl_3_) *δ* 7.55 (dd, *J* = 7.5, 1.9 Hz, 2H), 7.47–7.35 (m, 3H), 6.43 (d, *J* = 8.5 Hz, 2H), 6.23 (d, *J* = 8.6 Hz, 2H), 3.96 (t, *J* = 5.9 Hz, 2H), 3.71–3.56 (m, 10H), 3.50 (t, *J* = 5.3 Hz, 2H), 3.24 (q, *J* = 7.1 Hz, 4H), 2.86 (t, *J* = 5.1 Hz, 2H), 2.39 (br s, 2H), 1.54 (s, 3H), 1.09 (t, *J* = 7.0 Hz, 6H). ^13^C NMR (75 MHz, CDCl_3_) *δ* 168.3, 164.4, 160.2, 146.8, 129.8, 129.4, 129.3, 128.6, 128.1, 128.0, 113.6, 111.4, 73.6, 70.8, 70.5, 70.3, 67.8, 49.2, 44.5, 42.0, 12.6, 12.1 ppm. HRMS calc. for C_29_H_41_N_4_O_5_ [M+H]^+^ 525.3077, found 525.3075.

#### *N*-[4-[[2-[2-[2-(2-Aminoethoxy)ethoxy]ethoxy]ethyl](ethyl)amino]phenyl]-5-methyl-3-phenylisoxazole-4-carboxamide (4)

Synthesis as described for compound **3**, using *tert*-butyl [3-[4-(5-methyl-3-phenylisoxazole-4-carboxamido)phenyl]-6,9,12-trioxa-3-azatetradecan-14-yl]carbamate **11** (32.6 mg, 0.0546 mmol). Chromatography on an amino column (3:1 EtOAc/EtOH in *n*-hexane 20% → 50%) gave compound **4** (20.2 mg, 75%) as a colorless oil. ^1^H NMR (400 MHz, CDCl_3_) *δ* 7.70–7.62 (m, 2H), 7.57–7.48 (m, 3H), 7.09–7.01 (m, 3H), 6.65–6.51 (dt, 2H), 3.67–3.54 (m, 10H), 3.49 (t, *J* = 5.2 Hz, 2H), 3.44 (t, *J* = 6.3 Hz, 2H), 3.35 (q, *J* = 7.0 Hz, 2H), 2.84 (t, *J* = 5.2 Hz, 2H), 2.76 (s, 3H), 1.91 (s, 2H), 1.10 (t, *J* = 7.0 Hz, 3H). ^13^C NMR (101 MHz, CDCl_3_) *δ* 174.6, 160.0, 159.3, 145.5, 130.8, 129.4, 129.3, 128.3, 126.1, 122.1, 112.2, 111.6, 73.2, 70.8, 70.7, 70.7, 70.4, 68.9, 50.3, 45.62, 41.77, 13.2, 12.2 ppm. HRMS calc. for C_27_H_37_N_4_O_5_ [M+H]^+^ 497.2764, found 497.2766.

#### *N*-[2-[2-[2-(2-Aminoethoxy)ethoxy]ethoxy]ethyl]-4-(diethylamino)-*N*-(3,5-dimethylisoxazol-4-yl)benzamide (5)

Synthesis as described for compound **3**, using *tert*-butyl [1-[4-(diethylamino)phenyl]-2-(3,5-dimethylisoxazol-4-yl)-1-oxo-5,8,11-trioxa-2-azatridecan-13-yl]carbamate **7** (63.6 mg, 0.113 mmol). Chromatography on an amino column (3:1 EtOAc/EtOH in *n*-heptane 20% → 50%) gave compound **5** (24.1 mg, 46%) as a yellowish solid. ^1^H NMR (400 MHz, CDCl_3_) *δ* 7.16 (d, *J* = 9.0 Hz, 2H), 6.41 (d, *J* = 9.0 Hz, 2H), 3.98 (br s, 1H), 3.68 (br s, 3H), 3.57 (m, 8H), 3.48 (t, *J* = 5.2 Hz, 2H), 3.30 (q, *J* = 7.1 Hz, 4H), 2.84 (t, *J* = 5.2 Hz, 2H), 2.16 (s, 3H), 2.11 (s, 3H), 1.42 (br s, 2H), 1.11 (t, *J* = 7.1 Hz, 6H). ^13^C NMR (101 MHz, CDCl_3_) *δ* 171.2, 164.1, 158.0, 149.3, 130.2, 122.0, 120.4, 110.1, 73.6, 70.7, 70.6, 70.40, 70.41, 68.6, 49.3, 44.4, 41.9, 12.6, 11.0, 9.9 ppm. HRMS calc. for C_24_H_39_N_4_O_5_ [M+H]^+^ 463.2920, found 463.2917.

#### *tert*-Butyl [2-[4-(diethylamino)phenyl]-1-(5-methyl-3-phenylisoxazol-4-yl)-1-oxo-5,8,11-trioxa-2-azatridecan-13-yl]carbamate (6)

A 30% suspension of potassium hydride in mineral oil (48.1 mg, 0.360 mmol, 2.8 equiv) was dissolved in anhydrous THF (3.0 mL). *N*-[4-(diethylamino)phenyl]-5-methyl-3-phenylisoxazole-4-carboxamide **1** (44.5 mg, 0.127 mmol) was added and the reaction mixture was stirred at room temperature for 2 h. *N*-Boc-PEG_3_-Br (50.0 mg, 0.140 mmol, 1.1 equiv) was dissolved in anhydrous THF (1.5 mL) and the resulting solution was added dropwise to the stirred reaction mixture. The reaction mixture was stirred at room temperature for 21 h and after this, additional portions of potassium hydride (0.0100 g, 0.249 mmol, 1.8 equiv) and *N*-Boc-PEG_3_-Br (20.0 mg, 0.0561 mmol, 0.44 equiv) were added. After 44 h, the reaction was quenched by adding distilled water and extracted with EtOAc. The combined organic phases were dried with anhydrous Na_2_SO_4_, filtered, and concentrated on rotary evaporator. Chromatography on silica gel (EtOAc*/n*-hexane 20% → 80%) gave compound **6** (48.0 mg, 60%) as a clear oil. ^1^H NMR (300 MHz, CDCl_3_) *δ* 7.58–7.52 (m, 2H), 7.40 (d, *J* = 7.5 Hz, 3H), 6.42 (d, *J* = 8.4 Hz, 2H), 6.21 (d, *J* = 8.6 Hz, 2H), 5.01 (s, 1H), 3.96 (t, *J* = 6.0 Hz, 2H), 3.71–3.56 (m, 10H), 3.51 (t, *J* = 5.3 Hz, 2H), 3.30 (t, *J* = 5.4 Hz, 2H), 3.23 (q, *J* = 7.1 Hz, 4H), 2.39 (s, 3H), 1.43 (s, 9H), 1.08 (t, *J* = 7.0 Hz, 6H) ppm. HRMS calc. for C_34_H_49_N_4_O_7_ [M+H]^+^ 625.3601, found 625.3602.

#### *tert*-Butyl [1-[4-(diethylamino)phenyl]-2-(3,5-dimethylisoxazol-4-yl)-1-oxo-5,8,11-trioxa-2-azatridecan-13-yl]carbamate (7)

4-(Diethylamino)-*N*-(3,5-dimethylisoxazol-4-yl)benzamide **2** (70.0 mg, 0.244 mmol) and a 30% suspension of potassium hydride in mineral oil (49.0 mg, containing 14.7 mg of KH, 0.365 mmol, 1.5 equiv) were dissolved in dry DMF (2.5 mL), and the resulting mixture was cooled to 0 °C. *N*-Boc-PEG_3_-Br (99.5 mg, 0.268 mmol, 1.1 equiv) was dissolved in DMF (0.5 mL), the solution was added dropwise to the reaction mixture, and stirred at room temperature for 69 h. *N*-Boc-PEG_3_-Br (15.0 mg, 0.0421 mmol, 0.2 equiv) and KH (32.5 mg, containing 9.75 mg of KH, 0.243 mmol) were added to the reaction mixture and stirring was continued for 1 h. The temperature was increased to 80 °C and stirring continued for 2 h. After a total of 72 h, the reaction was quenched with distilled water. The aqueous phase was extracted with EtOAc. The combined organic phases were washed with water, dried with anhydrous Na_2_SO_4_, filtered, and concentrated on rotary evaporator. Chromatography on silica gel (EtOAc*/n*-heptane 20% → 80%) gave compound **7** (68.3 mg, 51%) as a colorless oil. ^1^H NMR (400 MHz, CDCl_3_) *δ* 7.17 (d, *J* = 8.6 Hz, 2H), 6.43 (d, *J* = 8.6 Hz, 2H), 5.03 (s, 1H), 3.99 (s, 1H), 3.70 (s, 3H), 3.58 (m, 8H), 3.52 (t, *J* = 5.2 Hz, 2H), 3.32 (q, *J* = 7.0 Hz, 6H), 2.17 (s, 3H), 2.12 (s, 3H), 1.43 (s, 9H), 1.13 (t, *J* = 7.1 Hz, 6H). ^13^C NMR (101 MHz, CDCl_3_) *δ* 171.2, 164.1, 158.0, 149.4, 130.2, 121.0, 120.5, 110.1, 79.3, 70.7, 70.6, 70.4, 70.4, 70.3, 68.7, 49.3, 44.4, 40.5, 28.5, 12.6, 11.0, 9.9 ppm. HRMS calc. for C_29_H_47_N_4_O_7_ [M+H]^+^ 563.3445, found 563.3443.

#### *tert*-Butyl [3-(4-nitrophenyl)-6,9,12-trioxa-3-azatetradecan-14-yl]carbamate (9)

*N*-Ethyl-4-nitroaniline **8** (50.0 mg, 0.301 mmol) was dissolved in anhydrous DMF (0.6 mL). Sodium hydride (13.0 mg, 0.331 mmol, 1.1 equiv) was added and the reaction mixture was stirred at 0 °C for 15 min until hydrogen evolution ceased. *N*-Boc-PEG_3_-Br (118 mg, 0.331 mmol, 1.1 equiv) was dissolved in anhydrous DMF (0.4 mL) and the solution was added dropwise to the stirred reaction mixture. The resulting mixture was stirred for 23 h at room temperature, quenched with distilled water and extracted with EtOAc. The combined organic phases were repeatedly washed with water, dried with anhydrous Na_2_SO_4_, filtered, and concentrated on rotary evaporator. Chromatography on silica gel (EtOAc*/n*-hexane 20% → 80%) gave compound **9** (53.9 mg, 41%) as a yellow oil. ^1^H NMR (400 MHz, CDCl_3_) *δ* 8.08 (d, *J* = 9.4 Hz, 2H), 6.63 (d, *J* = 9.4 Hz, 2H), 4.96 (s, 1H), 3.70–3.47 (m, 16H), 3.30 (q, *J* = 5.5 Hz, 2H), 1.43 (s, 9H), 1.21 (t, *J* = 7.1 Hz, 3H). ^13^C NMR (101 MHz, CDCl_3_) *δ* 156.1, 152.6, 126.5, 110.4, 79.4, 71.0, 70.7, 70.4, 68.7, 50.5, 46.2, 40.5, 28.6, 12.1 ppm. HRMS calc. for C_21_H_35_N_3_O_7_Na [M+Na]^+^ 464.2373, found 464.2372.

#### *tert*-Butyl [3-(4-aminophenyl)-6,9,12-trioxa-3-azatetradecan-14-yl]carbamate (10)

*tert*-Butyl [3-(4-nitrophenyl)-6,9,12-trioxa-3-azatetradecan-14-yl]carbamate **9** (74.3 mg, 0.168 mmol) was dissolved in a 1:1 mixture of EtOH and EtOAc (2 mL). The reaction mixture was placed under argon atmosphere, prior to addition of 10% Pd/C (~ 5 mg). A balloon filled with hydrogen was attached and after 22 h at room temperature, an additional portion of 10% Pd/C was added, and a new hydrogen balloon was attached. The reaction mixture was stirred for 6 h. The catalyst was removed by filtration over Celite and solvents were evaporated on rotary evaporator. Chromatography on amino column (EtOAc/*n*-hexane 20% → 50%) gave compound **10** (29.6 mg, 43%) as a brown oil. ^1^H NMR (400 MHz, CDCl_3_) *δ* 6.63 (s, 4H), 5.04 (s, 1H), 3.65–3.55 (m, 10H), 3.52 (t, *J* = 5.2 Hz, 2H), 3.42–3.21 (m, 8H), 3.32–3.23 (m, 4H), 1.43 (s, 9H), 1.08 (t, *J* = 7.0 Hz, 3H). ^13^C NMR (101 MHz, CDCl_3_) *δ* 156.1, 141.8, 137.5, 116.9, 115.7, 79.3, 70.8, 70.7, 70.7, 70.4, 70.3, 69.2, 51.2, 46.6, 40.5, 28.5, 12.4 ppm. HRMS calc. for C_21_H_38_N_3_O_5_ [M+H]^+^ 412.2811, found 412.2812.

#### *tert*-Butyl [3-[4-(5-methyl-3-phenylisoxazole-4-carboxamido)phenyl]-6,9,12-trioxa-3-azatetradecan-14-yl]carbamate (11)

*tert*-Butyl [3-(4-aminophenyl)-6,9,12-trioxa-3-azatetradecan-14-yl]carbamate **10** (29.6 mg, 0.0670 mmol) and 5-methyl-3-phenylisoxazole-4-carboxylic acid (13.6 mg, 0.0670 mmol) were dissolved in anhydrous DMF (1 mL) under argon. HATU (35.7 mg, 0.0939 mmol, 1.4 equiv) and Et_3_N (14.0 μL, 0.100 mmol, 1.5 equiv) were added and the resulting mixture was stirred at room temperature for 69 h. Water (10 mL) was added and the aqueous phase was extracted with Et_2_O. The combined organic phases were dried with anhydrous Na_2_SO_4_, filtered, and concentrated on rotary evaporator. Chromatography on silica gel (EtOAc/*n*-hexane 20% → 80%) gave compound **11** (33.0 mg, 83%) as a yellowish oil. ^1^H NMR (400 MHz, CDCl_3_) *δ* 7.70–7.62 (m, 2H), 7.58–7.51 (m, 3H), 7.06 (d, *J* = 8.8 Hz, 2H), 6.98 (s, 1H), 6.58 (d, *J* = 8.8 Hz, 2H), 5.00 (s, 1H), 3.65–3.54 (m, 10H), 3.52 (t, *J* = 5.2 Hz, 2H), 3.45 (t, *J* = 6.4 Hz, 2H), 3.35 (q, *J* = 7.0 Hz, 2H), 3.29 (q, *J* = 5.4 Hz, 2H), 2.76 (s, 3H), 1.43 (s, 9H), 1.10 (t, *J* = 7.0 Hz, 3H). ^13^C NMR (101 MHz, CDCl_3_) *δ* 174.6, 160.0, 159.2, 156.2, 145.4, 130.8, 129.4, 129.2, 128.3, 126.1, 122.0, 112.1, 111.6, 79.3, 70.8, 70.7, 70.7, 70.4, 70.3, 68.89, 50.27, 45.6, 40.5, 28.5, 13.2, 12.2 ppm. HRMS calc. for C_32_H_45_N_4_O_7_ [M+H]^+^ 597.3288, found 597.3289.

#### General procedure for compound immobilization

*N*-Hydroxysuccinimidyl-Sepharose® 4 Fast Flow was added into a plastic syringe with polymer filter and washed under suction by using Biotage® VacMaster™ manifold (Supplementary Fig. S8). Afterward, the syringe was closed with a stopper. The compound of interest was dissolved in DMF in small (generally three) portions and added to the syringe. The mixture was let to react at room temperature under shaking for 16–20 h. The solvent was drained with suction and a 50 mM solution of Tris hydrochloride in water (pH 8.5) was added to block remaining reactive moieties in Sepharose®. The mixture was let to react at room temperature under shaking for 2 h and the buffer solution was drained under suction. The Sepharose® was washed with a 50 mM solution of Tris hydrochloride in water (10 × 1 mL, pH 8.5), EtOH/H_2_O (1:4, 5 × 1 mL) and DCM (5 × 1 mL). The washed Sepharose® was dried *in vacuo*.

#### Pulldown probe (12)

Immobilization according to the General Procedure, using *N*-hydroxysuccinimidyl-Sepharose® 4 Fast Flow (1.2 mL, packed volume of moist gel in a syringe) and 2-[2-(2-methoxyethoxy)ethoxy]ethan-1-amine **S-1** (1.50 mg, 0.0092 mmol). The mixture was let to react under shaking at room temperature for 106 h.

#### Pulldown probe (13)

Immobilization according to the General Procedure, using *N*-hydroxysuccinimidyl-Sepharose® 4 Fast Flow (1.2 mL, packed volume of moist gel in a syringe) and *N*-[2-[2-[2-(2-aminoethoxy)ethoxy]ethoxy]ethyl]-4-(diethylamino)-*N*-(3,5-dimethylisoxazol-4-yl)benzamide **5** (23.4 mg, 0.0491 mmol). The mixture was let to react under shaking at room temperature for 20 h.

#### Pulldown probe (14)

Immobilization according to the General Procedure, using *N*-hydroxysuccinimidyl-Sepharose^®^ 4 Fast Flow (0.9 mL, packed volume of moist gel in a syringe) and *N*-[2-[2-[2-(2-aminoethoxy)ethoxy]ethoxy]ethyl]-*N*-[4-(diethylamino)phenyl]-5-methyl-3-phenylisoxazole-4-carboxamide **3** (9.00 mg, 0.0172 mmol). The mixture was let to react under shaking at room temperature for 16 h.

#### Pulldown probe (15)

Immobilization according to the General Procedure, using *N*-hydroxysuccinimidyl-Sepharose® 4 Fast Flow (1.2 mL, packed volume of moist gel in a syringe) and* N*-[4-[[2-[2-[2-(2-aminoethoxy)ethoxy]ethoxy]ethyl](ethyl)amino]phenyl]-5-methyl-3-phenylisoxazole-4-carboxamide **4** (19.4 mg, 0.0391 mmol). The mixture was let to react under shaking at room temperature for 20 h.

### Biological methods

#### Protein sample preparation for affinity chromatography

The COS-1 cell culture and plasmids expressing mouse GATA4 and NKX2-5 proteins have been described previously^15^. For the preparation of protein samples, the cells were seeded on a 6-well plate and transfected for 24 h with 0.6 µg of pMT2-GATA4 or pMT2-NKX2-5 using FuGENE 6 (Roche) reagent at 1:3 DNA:reagent ratio. Control samples were not transfected. To extract total proteins, the cells were lysed into non-denaturing lysis buffer (20 mM Tris–HCl, 150 mM NaCl, 1 mM EDTA, 1 mM EGTA, 1% Triton-X100, 2.5 mM sodium pyrophosphate, pH 7.5) with phosphatase inhibitors (1 mM β-glycerophosphate, 1 mM Na_3_VO_4_, 50 mM NaF) and protease inhibitors (Protease Inhibitor Mini Tablets, #88,666, Pierce). The cells were disrupted by vortexing vigorously for 20 s followed by centrifugation at 15,000 *g* for 20 min at 4 °C. The supernatant containing the total proteins was transferred into clean sample tube. The protein concentration was measured with the Bradford assay from samples diluted in water to reduce Triton-X100 interference with the assay.

#### Affinity chromatography

The affinity chromatography protocol was based on the immunoprecipitation protocol described in Kinnunen et al.^15^ After solid phase synthesis, compounds immobilized to Sepharose® were suspended into 20% ethanol. To approximate equal amount of Sepharose® in each sample, we used the quantity of dry and packed Sepharose® originally used in synthesis reactions corresponding to 15 µL of packed Sepharose® in affinity reactions. The reactions were done in 1.5 mL microcentrifuge tubes and kept on ice. To remove ethanol, Sepharose® was washed twice with the lysis buffer described above (without inhibitors) by gently inverting the tubes several times and collecting the Sepharose® by centrifugation at 10,000 *g* for 10 s at 4 °C. The binding reactions were performed overnight under gentle agitation at 4 °C with 30 µg of total protein lysate in 0.95 mL volume of lysis buffer with phosphatase inhibitors (0.2 mM Na_3_VO_4_, 50 mM NaF) and protease inhibitors. The samples were then washed gently three times with 0.7 mL of lysis buffer (with inhibitors). Next day, the samples were collected by centrifugation at 10,000 *g* for 15 s at 4 °C and the supernatant was removed. The samples were then washed three times with 0.7 mL of lysis buffer (with inhibitors) by gently inverting the sample tubes several times, letting the samples settle down for 3 min, centrifuging as described above and removing the supernatant between the washes. Finally, the samples were boiled in 20 µL of SDS sample buffer (62.5 mM Tris–HCl pH 6.8, 2% SDS, 10% glycerol, 1.25% 2-mercaptoethanol, 0.1% bromophenol blue) for 4 min and vortexed vigorously to elute the bound proteins from Sepharose®. A 21-µL sample of supernatant was analyzed with western blotting. The method optimization, whole blot images and quantifications are presented in Supplementary information (Supplementary Fig. S1–S4).

#### Alpha screen

The plasmids expressing human GATA4-C-V5 and NKX2-5-N-SH were gift from Dr. Markku Varjosalo (University of Helsinki, Finland). COS-1 cells were seeded onto 6-well plates at 300,000 cells per well and transfected next day with 3 µg pDEST40-GATA4-C-V5 or 2.4 µg pcDNA™5/FRT/TO-NKX2-5-N-SH using FuGene6 (Promega) in 3:1 ratio to DNA. After 24 h the cells were detached by trypsin and counted with hemocytometer. Medium was removed by centrifugation 200 *g* at 4 °C for 4 min and the cells were washed once with phosphate buffered saline (PBS). Finally, the cells were suspended into non-denaturing lysis buffer (20 mM Tris–HCl, 150 mM NaCl, 1 mM EDTA, 1 mM EGTA, 1% Triton-X100, 2.5 mM sodium pyrophosphate, pH 7.5) with phosphatase inhibitors (1 mM β-glycerophosphate, 1 mM Na_3_VO_4_, 50 mM NaF) and protease inhibitors (Protease Inhibitor Mini Tablets, #88,666, Pierce) to contain 4,000 cells per µL. The cells were disrupted by vortexing vigorously for 20 s followed by centrifugation at 15,000 *g* for 20 min at 4 °C. The supernatant containing the total proteins was transferred into clean sample tube. GATA4 and NKX2-5 protein lysates were diluted into Alpha Screen sample buffer (50 mM Tris–HCl pH7.4, 150 nM NaCl, 0.1% BSA) added to ½ AreaPlate-96 (#6,002,290, Perkin Elmer) in addition to dilution series of compound or DMSO into Alpha Screen sample buffer and incubated at 4 °C for 60 min. Each sample well contained proteins from 1,000 GATA4 overexpressed cells and 2,500 NKX2-5 overexpressed cells. V5-acceptor beads (AL129, Perkin Elmer) and Strep-Tactin donor beads (AS106, Perkin Elmer) were added 20 µg/mL in final concentration of each bead and incubated at room temperature covered from light for 60 min. The plate was red using Enspire Alpha plate reader (Perkin Elmer). The method optimization is presented in Supplementary information (Supplementary Fig. S5–S7).

#### CETSA

COS-1 cells were seeded onto 6-well plates at 400,000 cells per well and transfected next day with plasmids expressing wild type GATA4 or GATA4 containing the mutation R283A, as described in protein sample preparation, with the following exception: 0.3 µg of plasmids were used per well and the cells were transfected for 6 h. Medium containing the transfection reagent was removed and the cells were cultured in normal culturing medium for approximately 20 h before exposing to compound **1** (50 µM) or 0.1% DMSO for 1 h in cell culture incubator. The cells were washed with PBS and detached using trypsin. The cells from each well were collected into separate Eppendorf tubes and trypsin was inactivated with DMEM containing 10% fetal bovine serum. Subsequently, the samples were kept on ice. The cells were pelleted by centrifugation at 300 g for 3 min at 4 °C, washed once with cold PBS and lysed into 350 µl of non-denaturing lysis buffer containing protease inhibitors followed by vortexing and centrifugation (as described above). Supernatant containing the native total protein sample was aliquoted into 9 thin-walled PCR tubes 35 µl in each and set under thermal gradient (T100 Thermal Cycler, Bio-Rad). The cells were pre-warmed from 4 to 21 °C for 1 s, then heated into the target temperatures (37, 38.5, 41.3, 45.7, 51, 55.1, 58.1 or 60 °C) for 3.5 min and subsequently cooled at 25 °C for 2 min followed by cooling to 4 °C. For each sample set, one tube was kept on ice for 4 °C as for reference. Aggregated proteins were pelleted by centrifugation at 20,000 *g* for 20 min at 4 °C. Thirty microliters from each supernatant were transferred into clean tube with 10 µl of 4 × Laemmli sample buffer (Bio-Rad), boiled for 4 min and loaded on commercial 12% Mini-PROTEAN TGX Pre-Stained SDS-PAGE gel (Bio-Rad). GATA4 protein was analyzed by western blot method as described above with the exception of ChemiDoc MP Imaging System (Bio-Rad) used for imaging and Quantity One 4.6.6 (Bio-Rad) for image analysis. The experiment was repeated three times. The band intensities within each blot were compared to the reference sample that had been kept at 4 °C.

#### Computational methods

MOE 2019.01 software (Chemical Computing Group Inc, Canada) was utilized for the computational tasks such as sequence alignment, homology modeling, and ligand docking. A homology model for the C-terminal zinc finger of GATA4 was constructed using the highly conserved zinc finger domain GATA3 as a template structure (Protein Data Bank; 3DFX, sequence identity 76%). Sequence comparison did not contain any insertions or deletions. AMBER99 force field was applied for atom parametrization. Protein side chain rotamers were assembled from extensive rotamer library. During the homology modeling, 10 intermediate protein models were generated by Boltzmann-weighted randomized modeling procedure. Each intermediated model was refined until Root Mean Square (RMS) gradient drops below 1 by electrostatics-enabled protein minimization. Intermediate protein models were scored by using the Generalized Born/Volume integral (GB/VI) methodology. The highest ranked intermediate model was selected as a final model and further minimized until RMS drops below 0.5. Final model was inspected and confirmed to have adequate stereochemical quality with Ramachandran plots. Virtual ligand screening evaluations were carried out with docking application at MOE software. The broad binding cavity in C-terminal zinc finger of GATA4 protein model was characterized by Site Finder application as a probable ligand binding site. During the docking protocol, on-flight generated ligand conformations were placed in cavity with the Triangle Matcher method and ranked with the London dG scoring function by utilizing Amber10-Extended Hückel Theory-force field. Subsequently, 30 highest ranked poses were applied for refinement procedure containing the energy minimization and rescoring with the GBVI/WSA dG scoring function.

#### Statistics

Statistical analyses were performed in IBM SPSS Statistics software version 29. Statistical significance between two groups was evaluated by Levene’s test followed by independent samples t-test or Mann–Whitney U test. A *p*-value of < 0.05 was considered statistically significant.

### Supplementary Information

Supplementary Information.

## Data Availability

All data generated or analyzed during this study are included in this published article and its Supplementary information file.
