# Supplementary material for: Affinity chromatography reveals direct binding of the GATA4–NKX2-5 interaction inhibitor (3i-1000) with GATA4
Source: Sci Rep. 2024 Apr 18;14:8938. doi: 10.1038/s41598-024-59418-4 (PMC11026519; doi:10.1038/s41598-024-59418-4)
Supplement: Supplementary file 1 — Supplementary Information. [file 41598_2024_59418_MOESM1_ESM.docx]

**Supplementary information**

**Affinity chromatography reveals direct binding of the GATA4-NKX2-5 interaction inhibitor (3i-1000) with GATA4**

Mikael Jumppanen^1,+^, Sini M. Kinnunen^2,+^, Matej Zore^1,+^, Mika J. Välimäki^2^, Virpi Talman^2^, Gustav Boije af Gennäs^1^, Heikki J. Ruskoaho^2^, Jari Yli-Kauhaluoma^1,*^

^1^ Drug Research Program, Division of Pharmaceutical Chemistry and Technology, Faculty of Pharmacy, Viikinkaari 5 E (P.O. Box 56), FI-00014 University of Helsinki, Finland.
^2^ Drug Research Program, Division of Pharmacology and Pharmacotherapy, Faculty of Pharmacy, Viikinkaari 5 E (P.O. Box 56), FI-00014 University of Helsinki, Finland

* jari.yli-kauhaluoma@helsinki.fi

+ these authors contributed equally to this work

**Table of contents**

[Supplementary methods 2](#_Toc159943337)

[^1^H and ^13^C NMR spectra of compounds 11](#_Toc159943338)

[FTIR spectra of the pulldown probes 20](#_Toc159943339)

[References 21](#_Toc159943340)

# Supplementary methods

**Affinity chromatography method validation**

The method optimization was started by employing the same experimental conditions that we have used successfully in previous experiments showing GATA4 binding to NKX2-5-FLAG by agarose linked FLAG-antibody immunoprecipitation,^1^ i.e., overexpression of proteins in COS-1 cells, 30 µg of protein cell lysate per reaction, the same reaction buffers and volumes, incubation times, washing times and volumes, and the boiling the bound protein from Sepharose®. To verify sufficient washing of the unbound proteins in the current assay, 28 µL samples of the supernatant taken before washes and from the final third wash were subjected to Western blotting. We performed visual examination of total proteins based on Ponceau S (P7170, Sigma) staining and immunoblotting with GATA4 and NKX2-5 antibodies. Total proteins and GATA4 or NKX2-5 protein were detected before washes but not in the samples from the final wash, confirming that unbound proteins were successfully removed during the washing steps (Supplementary Fig. S1). Furthermore, affinity chromatography reactions with untransfected cell lysates showed no immunoreacitivity for GATA4 or NKX2-5 (Supplementary Fig. S1) validating specificity of the antibodies used. Ponceau S gave only faint red color and the membrane images were not illustrative (data not shown). Thus, silver staining was performed. The silver staining has much greater sensitivity than Ponceu S staining detecting even 2 ng protein^2^. Moreover, the color of the stain was more intensive so that the gel could be easily imaged. For the staining, a 28 µL samples taken before washes and from the final third wash were run on 10% SDS-PAGE gel. The gel was fixed for 15 min in a solution of 10% acetic acid and 30% EtOH and allowed to swell for 15 min in H_2_O by changing H_2_O one time. After this, the gel was soaked in freshly prepared Farmer’s reducer [10% K_3_Fe(CN)_6_, 16% Na_2_S_2_O_3_] for 2 min. The color was then removed by washing with H_2_O until the gel was clear. Next, the gel was placed in 0.1% AgNO_3_ for 30 min and washed with H_2_O for 15 s. Then the gel was placed in 2.5% Na_2_CO_3_ for 15 s and stained by adding 50 µL formaldehyde in 50 mL of 2.5% Na_2_CO_3_ until the proper intensity of staining was reached and then stopped the reaction by adding 2 mL acetic acid. The following day the gel was washed with H_2_O and placed in solution of 20% EtOH, 1.5% glycerol for 30 min. Finally, the gel was imaged with ChemiDoc MP Imaging System (Bio-Rad) (Supplementary Fig. S2).

**
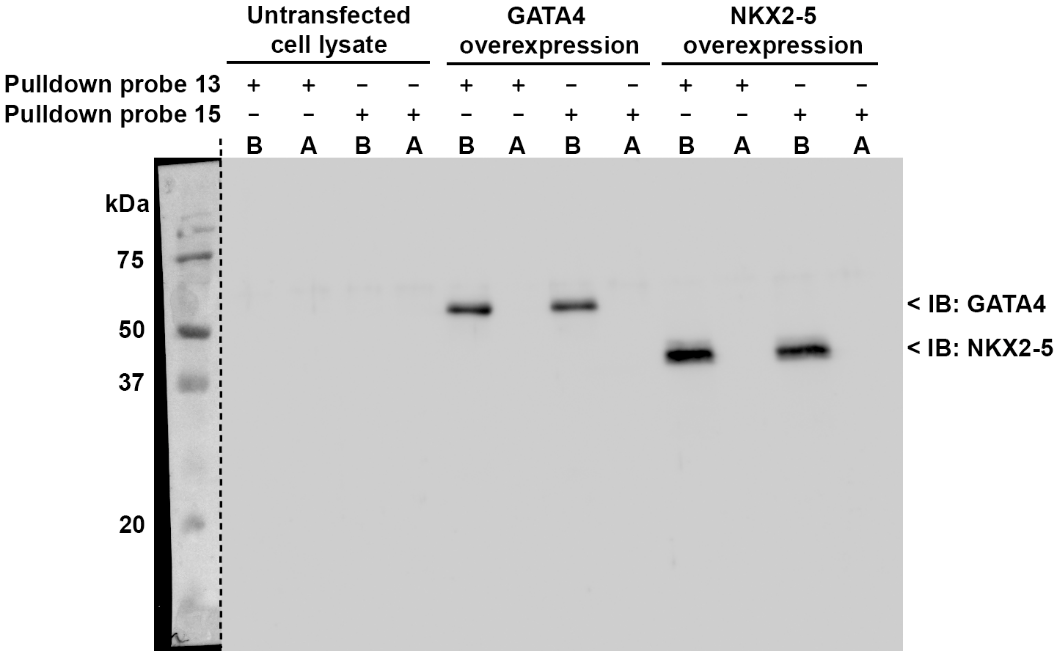
**

**Supplementary Figure S1.** Representative Western blot image of method validation showing sufficient washing of the unbound proteins and specificity of the antibodies used. The pulldown probes **13** and **15** were incubated overnight in a buffer containing total protein lysates from COS-1 cells with GATA4 or NKX2-5 overexpression or without overexpression. The samples for Western blot were taken from the supernatant before (B) and after (A) the washes. The membrane was first immunoblotted with GATA4 antibody to identify GATA4 immunoreactive bands and then with NKX2-5 antibody without stripping to visualize both proteins simultaneously. Samples: negative control pulldown probe **13** (inactive ligand); pulldown probe **15** (active ligand). IB, immunoblotted; kDa, kilodalton; B, sample taken before wash; A, sample taken after wash. The dashed line separates the overlayed chemiluminesence image and photograph taken at the same time to visualize the molecular weight markers in their correct position.


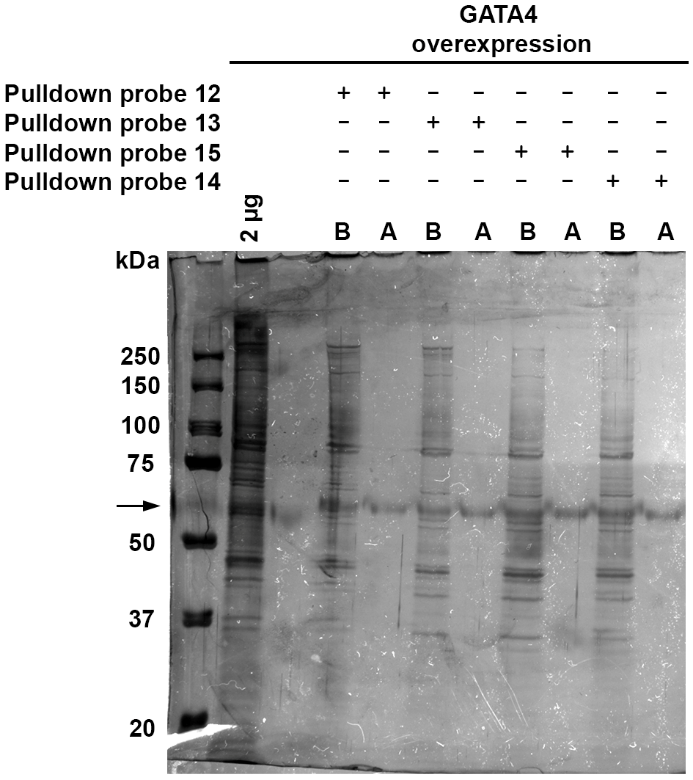


**Supplementary Figure S2.** Silver-stained SDS-PAGE gel of method validation showing sufficient washing of the unbound proteins. A 2 µg sample of protein lysate with GATA4 overexpression that was used in the affinity chromatography experiment. Samples: negative control pulldown probes **12** (PEG_3_ linker) and **13** (inactive ligand); pulldown probes **15** and **14** (active ligands). The arrow denotes an artefact appearing throughout the gel. B, sample taken before washes; A, sample taken after washes; kDa, kilodalton.

**Affinity chromatography western blot analysis of bound proteins**

The samples were resolved by SDS-PAGE and transferred onto nitrocellulose membrane (Amersham Protran Premium 10600004, GE Healthcare Life Sciences). The membranes were immunoblotted with GATA4 or NKX2-5 antibodies (sc-9053 and sc-8697, respectively, Santa Cruz Biotechnology, 1:1,000 dilution) and secondary antibodies (#7074, Cell Signaling Technology and sc-2020, Santa Cruz Biotechnology, both at 1:2,000). Pierce SuperSignal kit (#34078, Thermo Scientific) and Luminescent Image Analyzer LAS-3000 (Fuji) were used for visualization (Supplementary Fig. S3-S4).


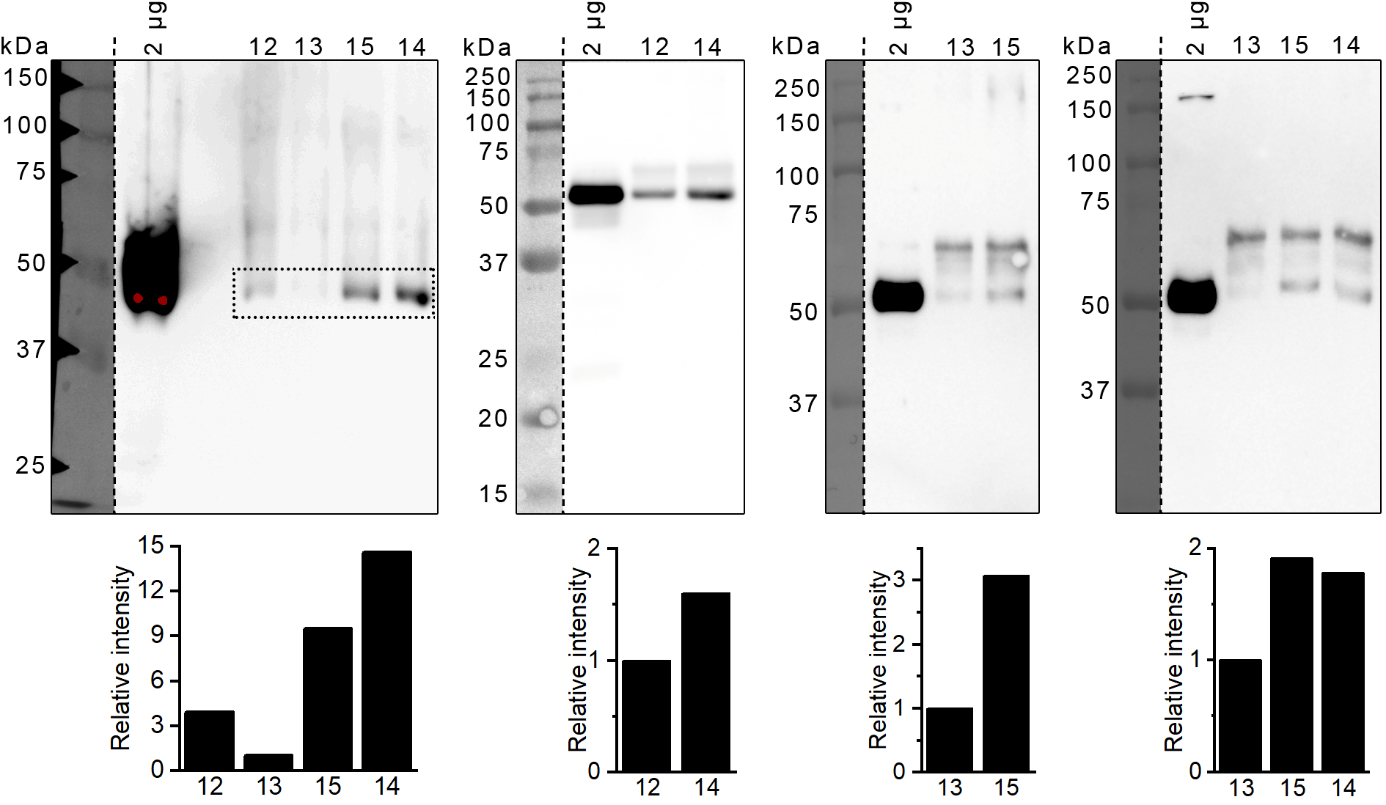


**Supplementary Figure S3.** Whole Western blot images and quantitations of GATA4 protein binding. 2 µg is a reference sample of protein lysate used in affinity chromatography experiment showing a 52 kDa band corresponding to GATA4. Blot corresponding to Fig. 3 is marked with dotted rectangular. Samples: negative control pulldown probes **12** (PEG_3_ linker) and **13** (inactive ligand); pulldown probes **15** and **14** (active ligands). kDa, kilodalton. The dashed line separates the overlayed chemiluminesence image and photograph taken at the same time to visualize the molecular weight markers in their correct position.


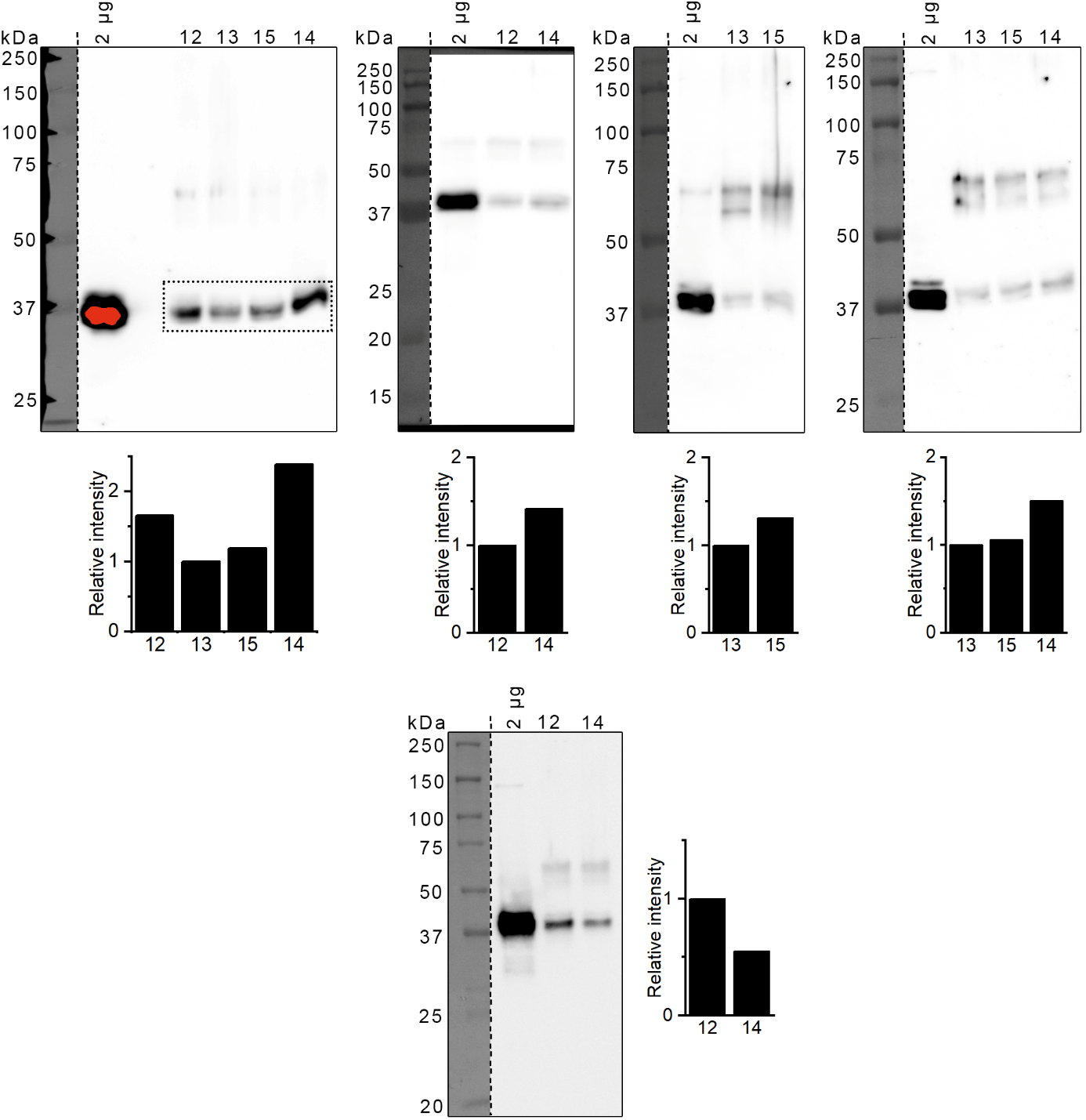


**Supplementary Figure S4.** Whole Western blot images and quantitations of NKX2-5 protein binding. A 2 µg reference sample of protein lysate used in affinity chromatography experiment showing a 37 kDa band corresponding to NKX2-5. Blot corresponding to Fig. 3 is marked with dotted rectangular. Samples: negative control pulldown probes **12** (PEG_3_ linker) and **13** (inactive ligand); pulldown probes **15** and **14** (active ligands). kDa, kilodalton. The dashed line separates the overlayed chemiluminesence image and photograph taken at the same time to visualize the molecular weight markers in their correct position.

**Alpha Screen method optimization**

To verify the protein expression levels of plasmids used in Alpha Screen, COS-1 cells were seeded onto 24-well plate at 70,000 cells per well and transfected with various amount of plasmids pMT2-GATA4, pDEST40-GATA4-C-V5, pMT2-NKX2-5 and pcDNA™5/FRT/TO-NKX2-5-N-SH as described above. After 24 hours the proteins were extracted using non-denaturing lysis buffer and analysed by Western blot as above. Supplementary Fig. S5a shows expression levels of pMT2-GATA4 (0.3 and 0.4 µg) and pDEST40-GATA4-C-V5 (0.5 and 1.0 µg). Supplementary Fig. S5b shows expression levels of pMT2-NKX2-5 (0.1 and 0.2 µg) and pcDNA™5/FRT/TO-NKX2-5-N-SH (0.3, 0.4, 0.5 and 1.0µg). The plasmid amount was then upscaled to 6-well plate.


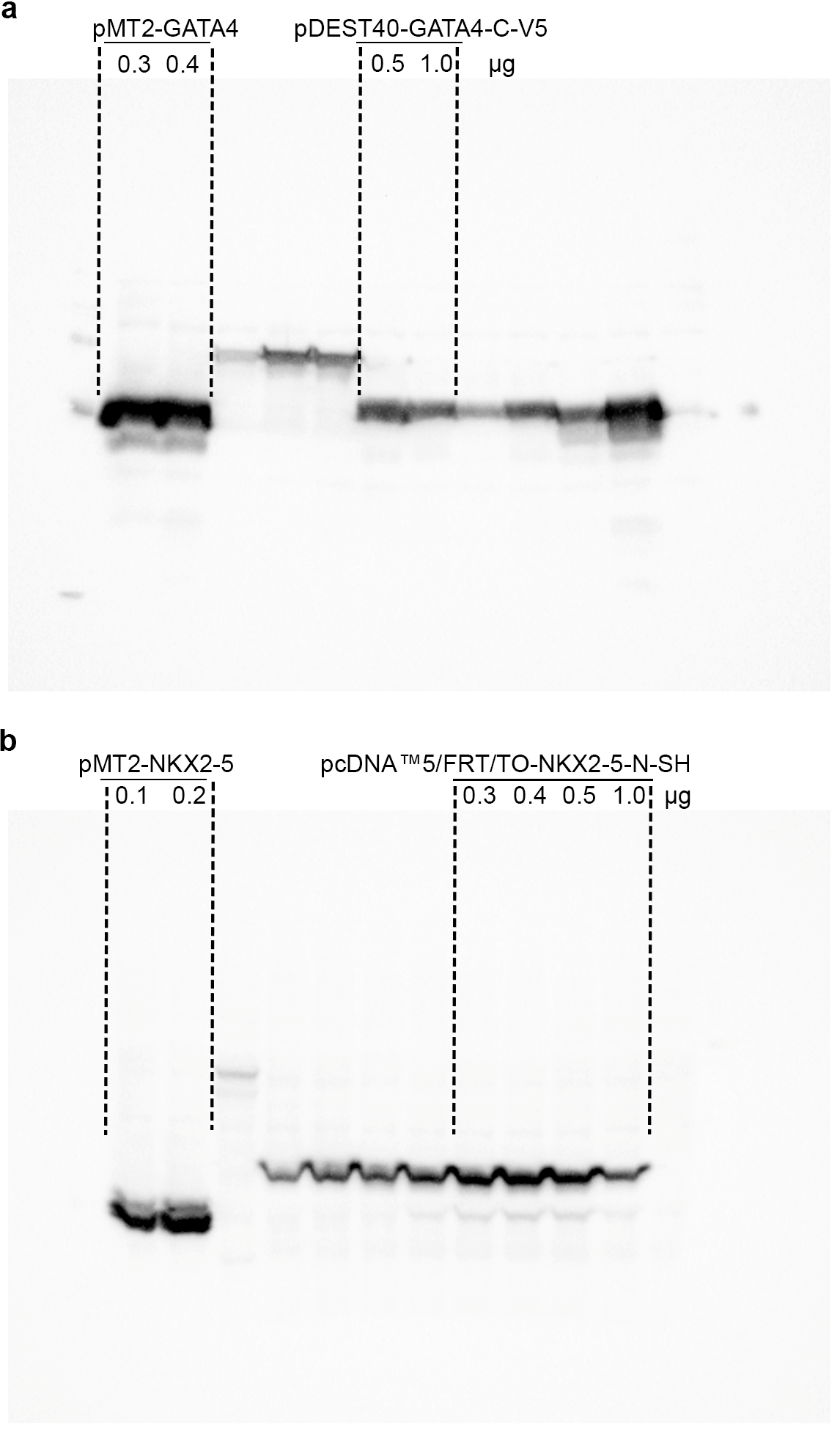


**Supplementary Figure S5.** Verification of protein expression of GATA4-C-V5 and NKX2-5-N-SH. COS-1 cells were transfected with various amount of plasmids, proteins were extracted to non-denaturing lysis buffer and western blot membranes were immunblotted with (**a**) anti-GATA4 (sc-9053) and (**b**) anti-NKX2-5 (sc-8697) antibodies. The pMT2-NKX2-5 contains mouse sequence of NKX2-5 and thus appears lower than human sequence of NKX2-5.

To optimize the protein lysis conditions, COS-1 cells were plated on 6-well plate 300,000 cells per well, transfected with 3 µg pDEST40-GATA4-C-V5 or 2.4 µg pcDNA™5/FRT/TO-NKX2-5-N-SH for 24 hours. The cells were trypsinized, calculated and washed with PBS. One sample set was lysed into Alpha SureFire Ultra Lysis Buffer (Perkin Elmer) with protease inhibitors and the other set was lysed into non-denaturing lysis buffer containing inhibitors as above. A protein cross-titration was performed keeping the concentrations of beads constant (20 μg/mL final concentration of each bead) and varying the concentration of each protein and keeping the end sample volume constant (40 µL). Proteins samples were diluted into Alpha Screen sample buffer (50 mM Tris-HCl pH7.4, 150 nM NaCl, 0.1% BSA) and added to sample plate to contain proteins of 20,000, 10,000 or 5,000 cells per well. Supplementary Fig. S6 shows that Alpha signal is higher with proteins extracted to non-denaturing lysis buffer (Fig. S6a) that to Alpha-lysis buffer (Fig. S6b).


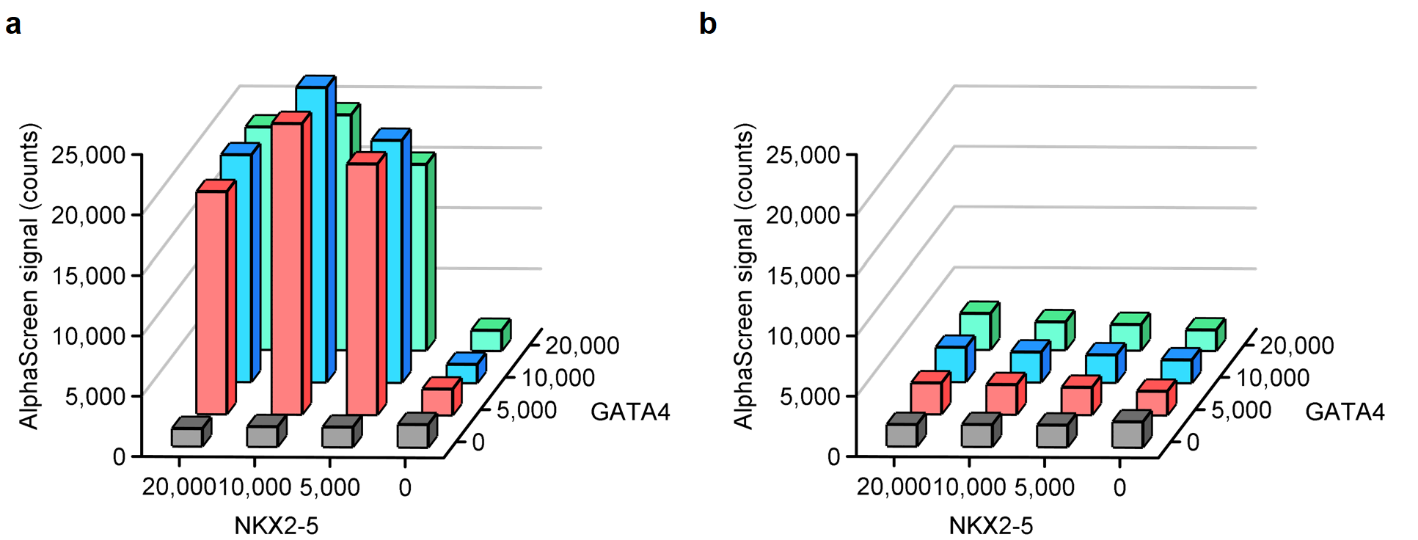


**Supplementary Figure S6.** Optimization of the protein lysis conditions. COS-1 cells overexpressing GATA4-C-V5 or NKX2-5-N-SH proteins were extracted to (**a**) non-denaturing lysis buffer or to (**b**) Alpha SureFire Ultra Lysis Buffer. The proteins in non-denaturing lysis buffer produces higher alpha signal counts. Number of experiments n=1.

Another set of protein cross-titration was performed testing the lower concentrations of proteins lysed into non-denaturing lysis buffer and retaining the concentrations of beads constant as above. Supplementary Fig. S7 shows the Alpha signal intensities including with the previous optimization data (Supplementary Fif. S6a). The protein concentrations which gives alpha signal on linear range turned out to be the most sensitive for compound testing i.e. GATA4 proteins from 1,000 cells and NKX2-5 proteins from 2,500 cells. The highest “hook point” signal for protein-protein interaction (24,000 counts with GATA4 proteins from 5,000 cells and NKX2-5 proteins from 5,000 cells) was the saturated condition and showed compound inhibition only with the highest compound concentrations (Supplementary Fig. 6b). In addition, NKX2-5-SH alone without GATA4-C-V5 protein produced only background Alpha signal (Supplementary Fig. 6b).

**
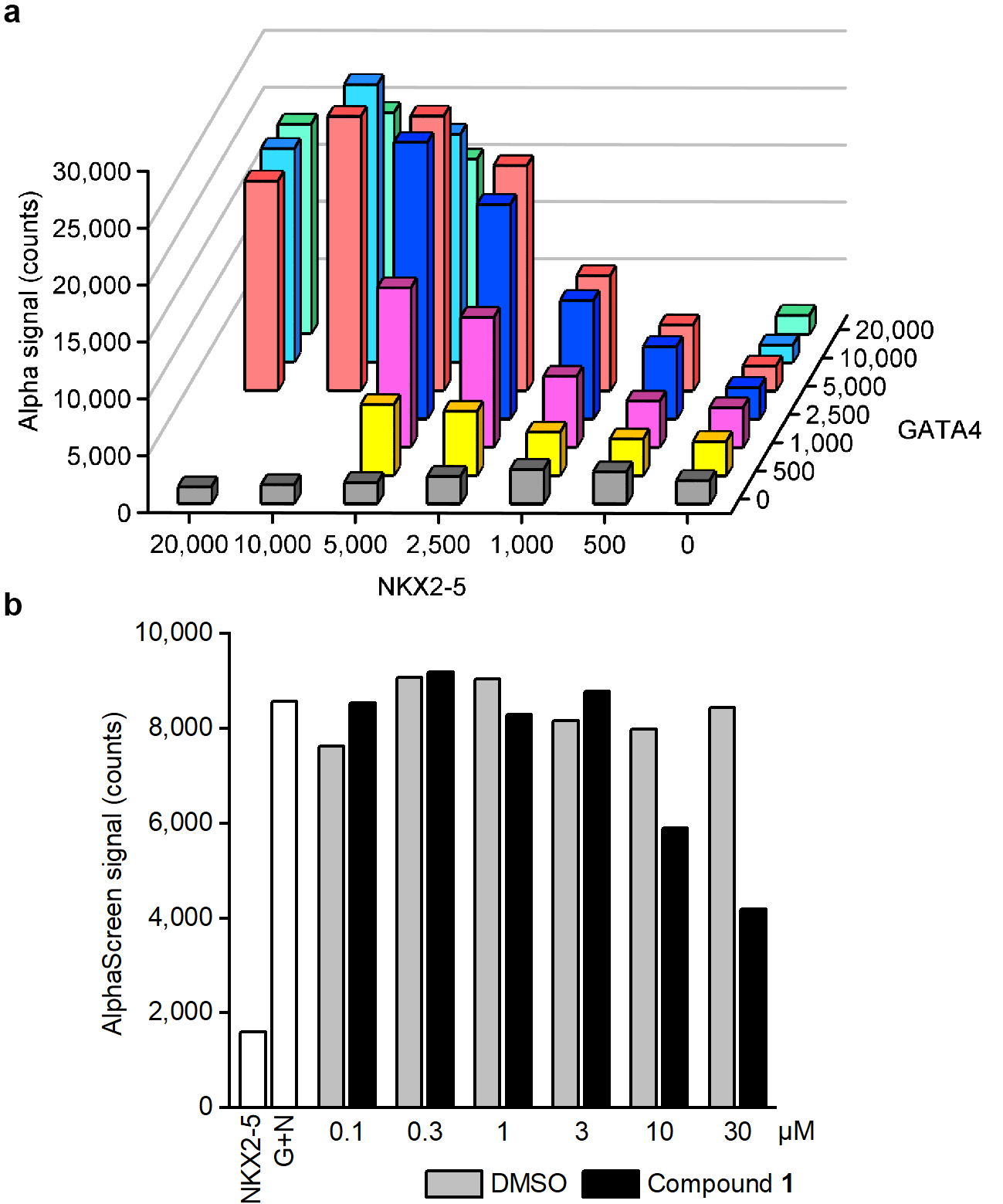
**

**Supplementary Figure S7.** Optimization of the protein concentrations. (**a**) Protein cross-titration was performed to achieve optimal alpha signal. Concentration giving alpa signal 11,000 counts (GATA4 1,000 and NKX2-5 2,500) was considered to be on linear range and selected for further tests. Number of experiments n=1, exept GATA4 5,000 and NKX2-5 5,000 is the average of 2 experiments. (**b**) The highest alpha signal with GATA4 proteins from 5,000 cells and NKX2-5 proteins from 5,000 cells is not enough sensitive to show compound inhibition with lower concentrations. n=1

**GATA4 homology model vs. GATA4 AlphaFold model**

The quality of GATA4 homology model presented in this manuscript was assessed and cross-validated through comparison to AlphaFold predicted GATA4 structure (AF-P43694-F1).^3^ The pairwise amino acid matrix for C-terminal zinc finger of GATA4 homology model shows a sound α-carbon correspondence with 0.798 Å RMSD in contrast to human GATA4 AlphaFold model. We assume that DNA-bound GATA4 model represents a most relevant protein conformation for computational assessment. The detailed superposition report regarding the GATA4 homology model and GATA4 AlphaFold protein (AF-P43694-F1) is presented in Supplementary Table 1.

**Supplementary Table 1.** Detailed superposition report regarding the GATA4 homology model presented in this study and GATA4 AlphaFold protein (AF-P43694-F1).

Superposition Report

Tue Feb 8 12:28:04 2022 (MOE 2020.09)

Options:

Atoms Used: CA

Pairwise RMSD Matrix:

Chains 1 4

1:AF-P43694-F1 0.80

4:Model 0.80

Overall RMSD: 0.798 A

1: AF-P43694-F1-model_v2-4.A

vs.

4: Model

RMSD = 0.798 A

ARG 266 - ARG 266 : 0.891

VAL 267 - VAL 267 : 1.175

GLY 268 - GLY 268 : 1.716

LEU 269 - LEU 269 : 1.638

SER 270 - SER 270 : 0.537

CYS 271 - CYS 271 : 0.200

ALA 272 - ALA 272 : 0.538

ASN 273 - ASN 273 : 0.695

CYS 274 - CYS 274 : 0.679

GLN 275 - GLN 275 : 0.442

THR 276 - THR 276 : 0.841

THR 277 - THR 277 : 0.803

THR 278 - THR 278 : 0.711

THR 279 - THR 279 : 0.882

THR 280 - THR 280 : 1.217

LEU 281 - LEU 281 : 0.864

TRP 282 - TRP 282 : 0.281

ARG 283 - ARG 283 : 0.408

ARG 284 - ARG 284 : 0.391

ASN 285 - ASN 285 : 0.434

ALA 286 - ALA 286 : 0.565

GLU 287 - GLU 287 : 0.495

GLY 288 - GLY 288 : 0.218

GLU 289 - GLU 289 : 0.506

PRO 290 - PRO 290 : 0.664

VAL 291 - VAL 291 : 0.373

CYS 292 - CYS 292 : 0.503

ASN 293 - ASN 293 : 0.817

ALA 294 - ALA 294 : 0.902

CYS 295 - CYS 295 : 0.790

GLY 296 - GLY 296 : 0.834

LEU 297 - LEU 297 : 0.753

TYR 298 - TYR 298 : 0.558

MET 299 - MET 299 : 0.565

LYS 300 - LYS 300 : 0.764

LEU 301 - LEU 301 : 0.450

HIS 302 - HIS 302 : 0.555

GLY 303 - GLY 303 : 0.385

VAL 304 - VAL 304 : 0.732

PRO 305 - PRO 305 : 0.364

ARG 306 - ARG 306 : 0.557

PRO 307 - PRO 307 : 0.609

LEU 308 - LEU 308 : 0.564

ALA 309 - ALA 309 : 0.296

MET 310 - MET 310 : 0.264

ARG 311 - ARG 311 : 0.144

LYS 312 - LYS 312 : 0.869

GLU 313 - GLU 313 : 1.643

GLY 314 - GLY 314 : 1.498

ILE 315 - ILE 315 : 1.302

GLN 316 - GLN 316 : 0.709

THR 317 - THR 317 : 0.370

ARG 318 - ARG 318 : 0.599

LYS 319 - LYS 319 : 1.470


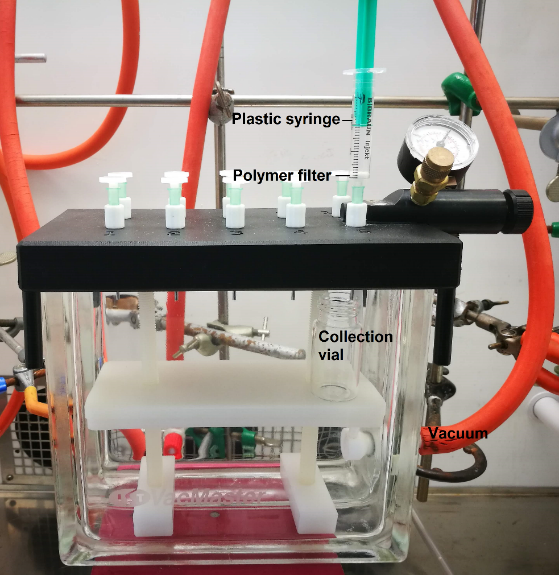


**Supplementary Figure S8.** Experimental setup for compound immobilization.

# ^1^H and ^13^C NMR spectra of compounds

**4-(Diethylamino)-*N*-(3,5-dimethylisoxazol-4-yl)benzamide (2)**

***N*-[2-[2-[2-(2-Aminoethoxy)ethoxy]ethoxy]ethyl]-*N*-[4-(diethylamino)phenyl]-5-methyl-3-phenylisoxazole-4-carboxamide (3)**

***N*-[4-[[2-[2-[2-(2-Aminoethoxy)ethoxy]ethoxy]ethyl](ethyl)amino]phenyl]-5-methyl-3-phenylisoxazole-4-carboxamide (4)**

***N*-[2-[2-[2-(2-Aminoethoxy)ethoxy]ethoxy]ethyl]-4-(diethylamino)-*N*-(3,5-dimethylisoxazol-4-yl)benzamide (5)**

***tert*-Butyl [2-[4-(diethylamino)phenyl]-1-(5-methyl-3-phenylisoxazol-4-yl)-1-oxo-5,8,11-trioxa-2-azatridecan-13-yl]carbamate (6)**

***tert*-Butyl [1-[4-(diethylamino)phenyl]-2-(3,5-dimethylisoxazol-4-yl)-1-oxo-5,8,11-trioxa-2-azatridecan-13-yl]carbamate (7)**

***tert*-Butyl [3-(4-nitrophenyl)-6,9,12-trioxa-3-azatetradecan-14-yl]carbamate (9)**

***tert*-Butyl [3-(4-aminophenyl)-6,9,12-trioxa-3-azatetradecan-14-yl]carbamate (10)**

***tert*-Butyl [3-[4-(5-methyl-3-phenylisoxazole-4-carboxamido)phenyl]-6,9,12-trioxa-3-azatetradecan-14-yl]carbamate (11)**

# FTIR spectra of the pulldown probes

**
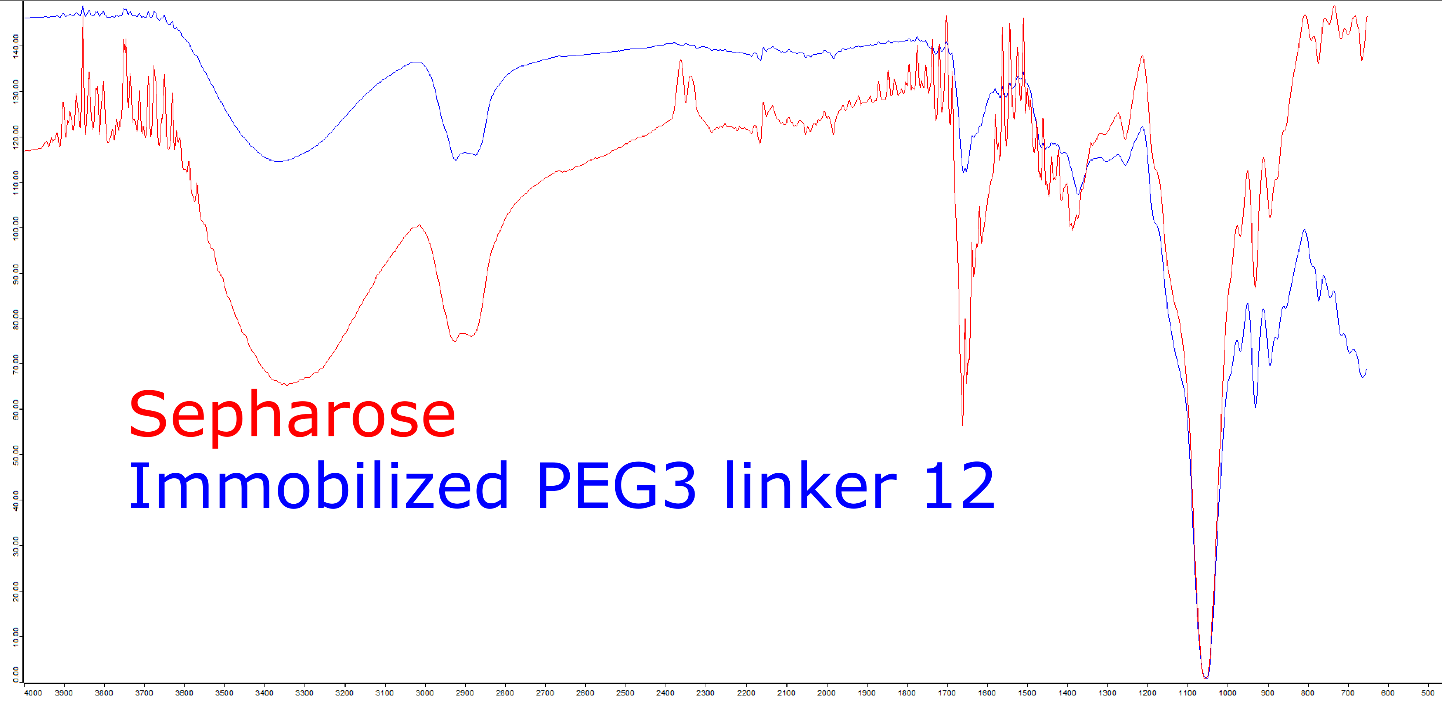
**

**
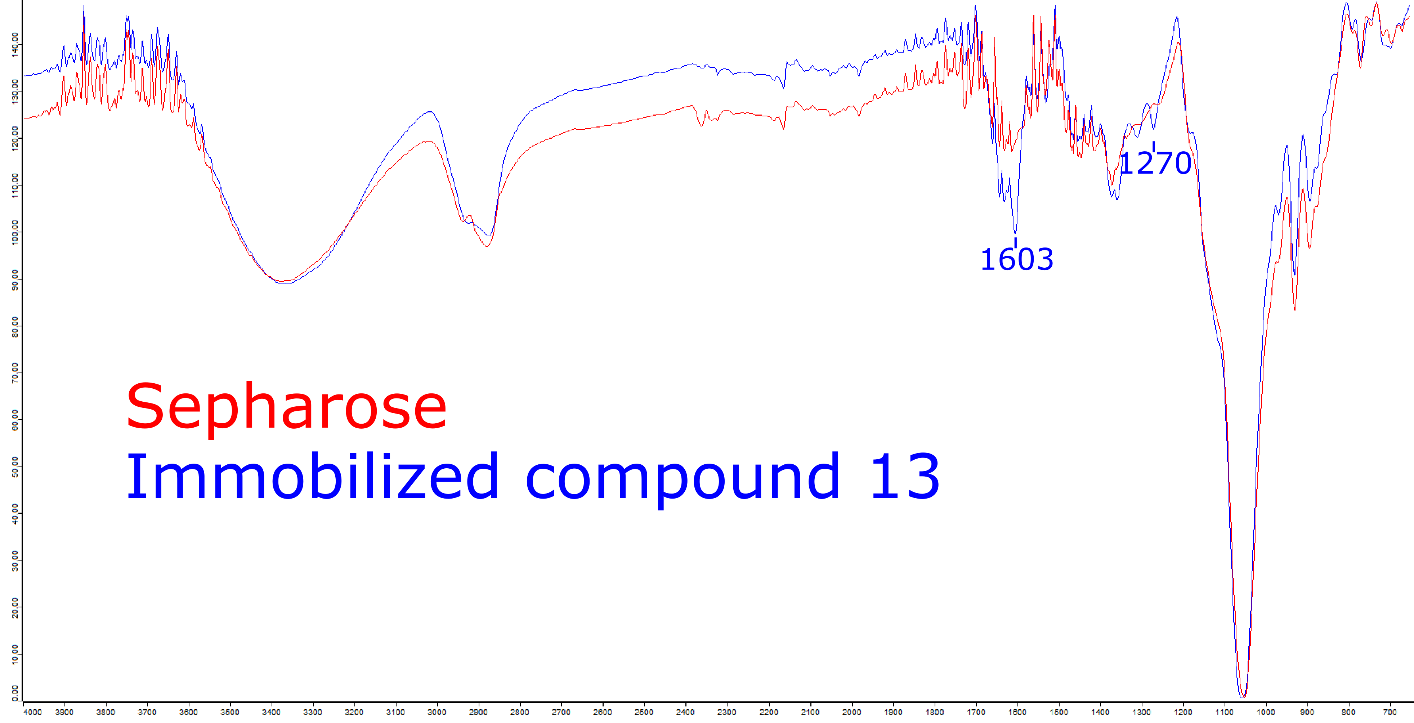
**

**
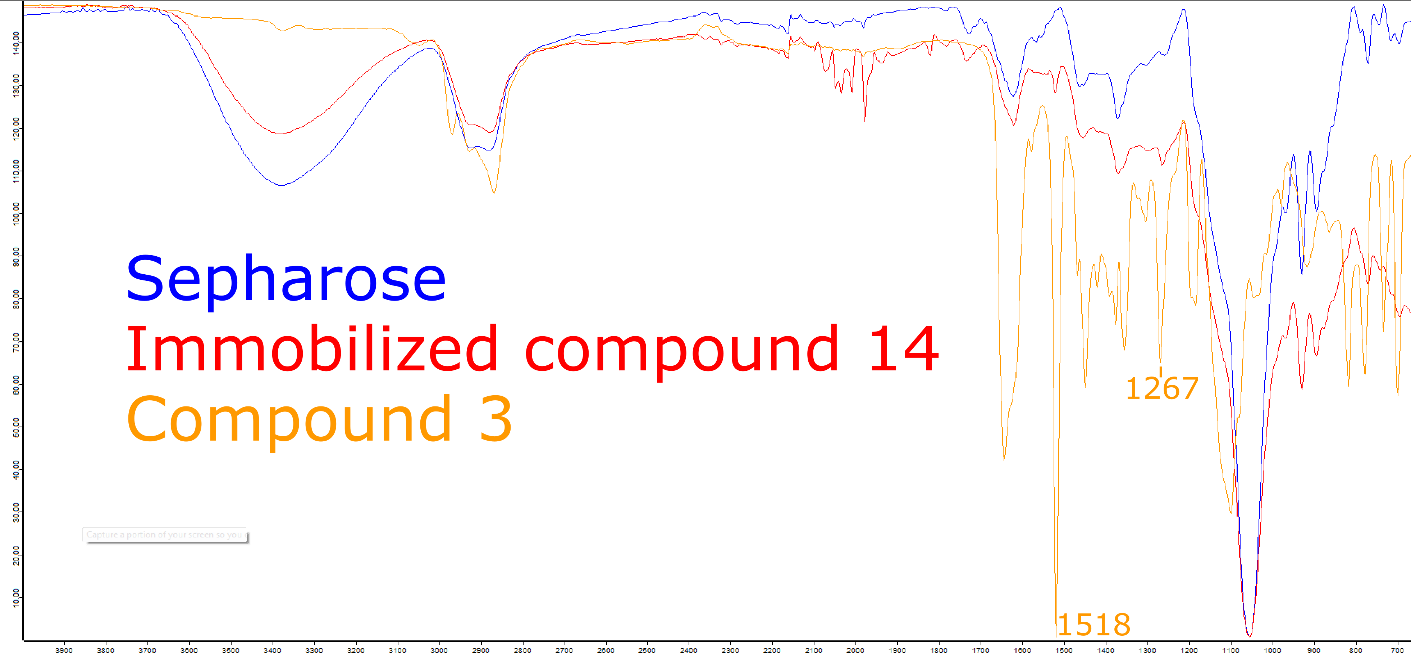
**

**
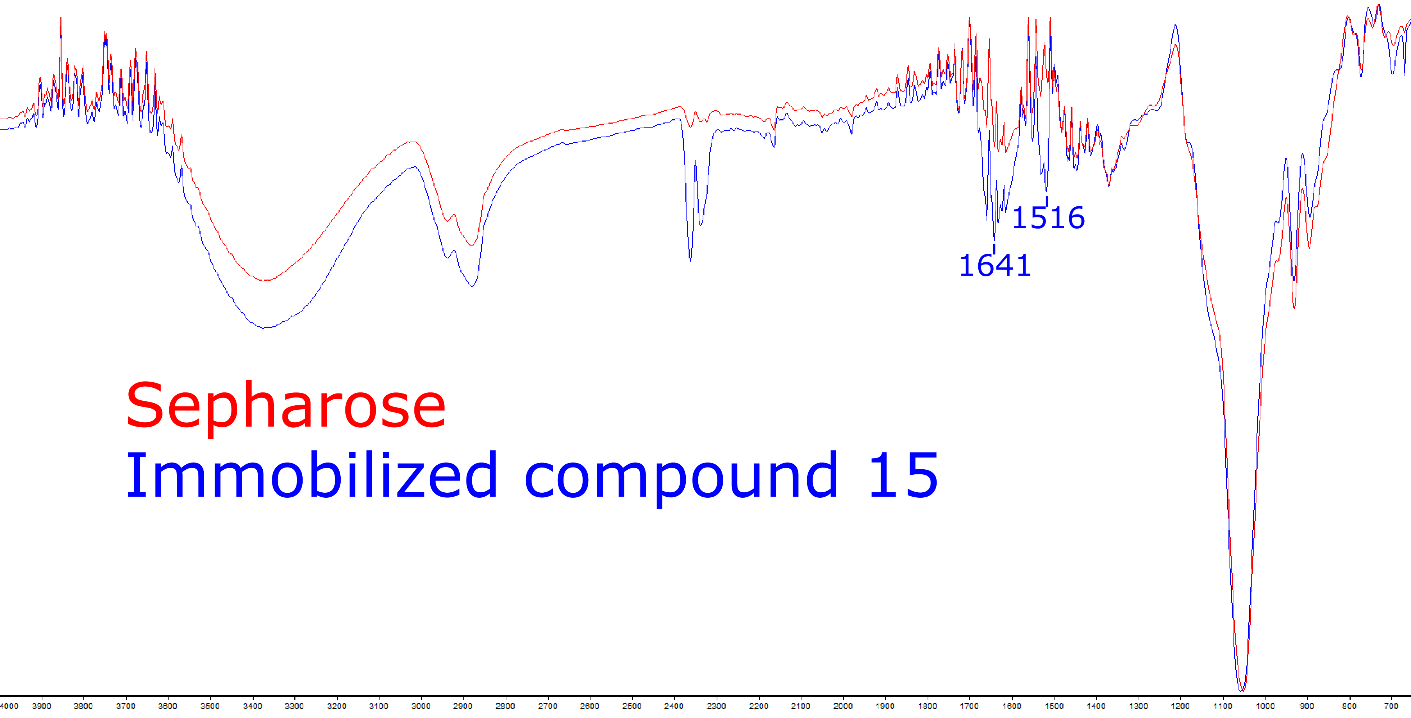
**

# References

1. Kinnunen, S. *et al.* Nuclear Receptor-Like Structure and Interaction of Congenital Heart Disease-Associated Factors GATA4 and NKX2-5. *PLoS One* **10**, e0144145 (2015).

2. Corley, R. B. *A Guide to Methods in the Biomedical Sciences: Identification of proteins in gels by staining.* (Springer Science + Business Media, Inc., 2006).

3. Jumper, J. *et al.* Highly accurate protein structure prediction with AlphaFold. *Nature* **596**, 583–589 (2021).
